# Recent Advances in Performance-Driven Multiscale Strategies for Epoxy-Based Underfill Materials for Advanced Packaging

**DOI:** 10.3390/polym18202481

**Published:** 2026-10-11

**Authors:** Yi Wang, Yage Wang, Yihang Wu, Yujie Yan, Yuechan Li, Dongya Sun, An Xie

**Affiliations:** Key Laboratory of Functional Materials and Applications of Fujian Province, School of Materials Science and Engineering, Xiamen University of Technology, Xiamen 361024, China

**Keywords:** electronic packaging, epoxy resin, flip-chip packaging, underfill encapsulation

## Abstract

The rapid evolution of advanced packaging toward high-density, high-power, and three-dimensional heterogeneous integration has placed increasingly stringent demands on package reliability, particularly because of the thermal expansion mismatch and associated thermomechanical stresses between silicon dies and substrates. Owing to their favorable combination of properties and highly tunable formulation chemistry, epoxy resins remain the predominant matrix materials for underfill applications. However, the design paradigm of epoxy-based underfills is shifting from the optimization of individual thermomechanical properties toward the coordinated regulation of thermal, rheological, mechanical, and reliability-related characteristics. This review summarizes recent advances in epoxy-based underfill materials from the perspectives of thermal management, rheological optimization, mechanical reinforcement, and reliability enhancement, with particular emphasis on the roles of filler engineering, resin and interfacial design, and curing-system regulation. The intrinsic coupling and trade-offs among different performance parameters are also discussed. Furthermore, emerging directions for underfill materials in advanced packaging are outlined, including multiscale structure–property relationships, reliability prediction, and intelligent formulation design. This review aims to provide insights into the synergistic design and practical implementation of high-performance epoxy-based underfill materials for next-generation electronic packaging.

## 1. Introduction

Driven by the rapid advancement of artificial intelligence (AI), high-performance computing, and high-speed communication, semiconductor devices are undergoing a transformative shift from conventional two-dimensional integration toward increasingly dense, multifunctional, three-dimensional heterogeneous architectures. In the post-Moore era, advanced packaging has emerged as a pivotal pathway for sustaining the continued improvement of system-level computing performance. Among the diverse packaging technologies available, flip-chip (FC) packaging has been widely adopted in high-performance processors, AI accelerators, and radio-frequency (RF) devices, owing to its high input/output (I/O) density, short interconnect lengths, and superior electrical performance. Nevertheless, as chip dimensions continue to grow, interconnect pitches shrink, and power densities escalate, thermomechanical reliability has become a critical challenge that may fundamentally constrain the further evolution of advanced packaging.

A fundamental reliability concern in flip-chip assemblies arises from the pronounced mismatch in thermal expansion among the constituent materials. The coefficient of thermal expansion (CTE) of silicon is approximately 2.6 ppm/°C, whereas that of typical organic substrates generally falls within 14–24 ppm/°C [1,2]. Under repeated temperature excursions, the resulting differential deformation generates cyclic thermomechanical stresses that become concentrated around microbumps and solder joints, potentially leading to fatigue cracking, interfacial delamination, and electrical failure. Effective redistribution and relaxation of these thermomechanical stresses are therefore essential for maintaining the long-term reliability of flip-chip packages.

**Figure 1:**
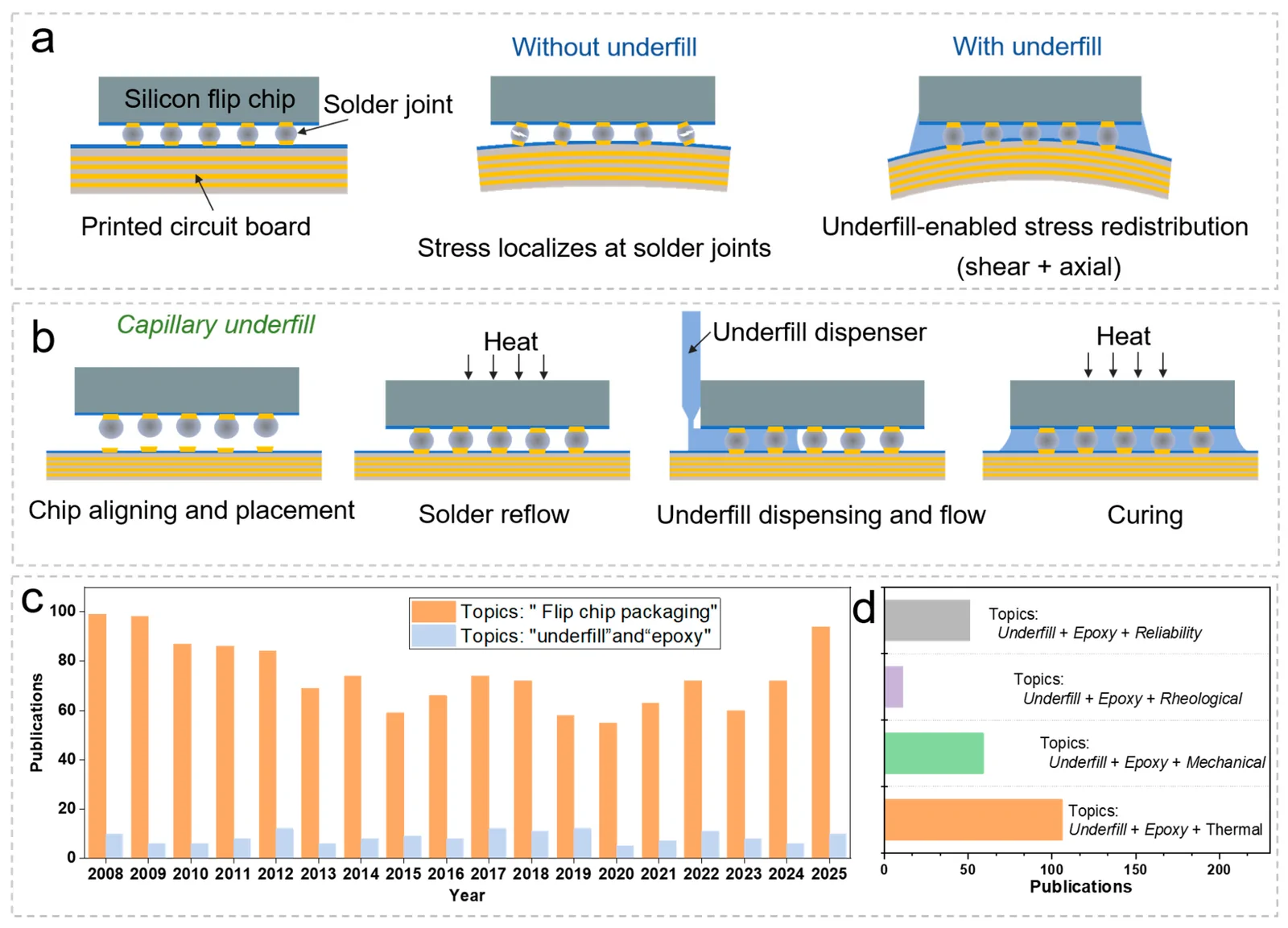
(**a**) Stress distribution in flip-chip packages without and with underfill, where the underfill redistributes mismatch stress from solder joints to the entire chip–substrate assembly. (**b**) Capillary underfill process: placement, reflow, dispensing, and curing. (**c**) Annual publications (2008–2025) from Web of Science Core Collection. (**d**) Publication distribution across four sub-topics of epoxy underfill research: thermal (106) dominates, followed by mechanical (59), with rheological (51) and reliability (11) considerably underexplored.

Underfill materials provide an effective and versatile solution to these reliability challenges. By filling the microscale gap between the die and the substrate—typically on the order of tens of micrometers—an underfill redistributes the thermomechanical loads that would otherwise concentrate at the interconnects across a larger portion of the package. In doing so, it simultaneously enhances resistance to moisture ingress, mechanical impact, and fatigue damage (Figure 1a). Properly engineered underfill systems have been demonstrated to substantially extend the thermal-cycling lifetime of flip-chip packages [3]. Depending on the filling process, underfill technologies can be broadly categorized as capillary underfill (CUF), no-flow underfill (NUF), molded underfill (MUF), and wafer-level underfill (WLUF). Among these approaches, CUF remains the most widely implemented in advanced flip-chip packaging because of its mature processing technology, compatibility with existing manufacturing infrastructure, and well-established reliability. A representative CUF process is illustrated in Figure 1b.

Among the various polymeric systems investigated for underfill applications, epoxy resins have become the predominant matrix materials in industry, owing to their excellent adhesion, dimensional stability, chemical resistance, and tunable crosslinked structures. However, as packaging technologies continue to advance toward higher power density, higher operating frequency, and greater miniaturization, the functional role of underfills has expanded well beyond mitigating thermal expansion mismatch—now encompassing heat dissipation, mechanical reinforcement, and environmental protection as well. Consequently, optimizing a single property is no longer sufficient to meet the increasingly complex demands of advanced packaging, and underfill design is progressively shifting toward multidimensional, synergistic property regulation. From a materials-design perspective, these requirements are inherently coupled and often conflicting. Reducing the CTE generally necessitates a high loading of low-expansion inorganic fillers; yet increasing the filler content can markedly elevate viscosity and compromise processability. Likewise, enhancing thermal conductivity typically requires the formation of interconnected heat-transfer pathways, whereas highly thermally conductive fillers are often relatively rigid and may undermine the toughness of the resulting composite. The optimization of underfill materials is therefore intrinsically a multivariable problem, governed by the coupled effects of resin structure, filler engineering, interfacial interactions, and processing conditions.

Bibliometric analysis further reveals that research on underfill materials remains relatively fragmented. According to the Web of Science Core Collection, 1342 publications were identified between 2008 and 2025 using “flip chip packaging” as the topic, whereas only 155 publications were retrieved using the combined topics “underfill” and “epoxy” (Figure 1c). This disparity suggests that, although underfill is an integral component of advanced packaging research, systematic investigations specifically focused on epoxy-based underfills remain comparatively limited. In terms of research focus, existing studies have largely emphasized thermal properties, package reliability, and filler-induced reinforcement, whereas systematic investigations into rheological behavior, interfacial regulation, and the mechanisms governing the synergy among multiple properties are still relatively scarce (Figure 1d). Consequently, much of the existing literature has concentrated either on individual material properties or on package-level performance, while the underlying relationships linking resin structure, filler organization, interfacial interactions, and processing behavior remain insufficiently understood.

Existing reviews on underfill materials have primarily addressed package reliability, thermal-conductivity enhancement, and filler selection. Zhang et al. [4] provided a systematic overview of the interplay among underfill materials, packaging processes, and reliability. Wen et al. [3] reviewed the development of thermally conductive epoxy-based underfills and discussed strategies for balancing their thermal, mechanical, electrical, and processing characteristics. From the perspective of filler classification, Cheong et al. [5] examined the effects of natural and synthetic fillers on the wetting and thermal properties of underfill systems. Plachý et al. [6] further highlighted the trade-off between reliability and processability in underfill material selection. Nevertheless, these reviews have generally been organized around specific properties, filler systems, or packaging processes, while the relationships and trade-offs among different performance requirements have received comparatively less systematic discussion. In particular, the connections among thermal, rheological, mechanical, and reliability-related properties, as well as the roles of resin molecular structure, filler architecture, interfacial characteristics, polymer-network structure, and curing history, warrant further consideration from a materials-design perspective. To address this gap, this review establishes a performance-oriented design framework for epoxy-based underfill materials by organizing recent advances around four key performance dimensions: thermal regulation, rheological optimization, mechanical reinforcement, and reliability enhancement. The discussion further considers the coupling and trade-offs among these properties and relates them to molecular structure, filler and interfacial design, polymer-network engineering, and processing history. Particular attention is given to how filler structural design, interfacial chemical regulation, polymer-network engineering, and cross-scale synergistic strategies govern the resulting material properties [7]. The coupling and trade-offs among different performance parameters are further discussed, together with emerging research directions, with the aim of providing a materials-design basis for the development of next-generation epoxy-based underfills with enhanced performance and reliability.

## 2. Multidimensional Performance Requirements of Epoxy-Based Underfill Materials

Underfill materials perform multiple functions within an electronic package, including thermomechanical stress redistribution, protection of interconnects, heat dissipation, and environmental barrier protection. Their performance requirements are therefore inherently multidimensional and strongly coupled. An ideal underfill system must possess appropriate rheological and wetting characteristics to enable rapid and complete penetration into microscale gaps while maintaining strong interfacial adhesion. At the same time, it must provide a suitable combination of thermal conductivity, coefficient of thermal expansion (CTE), elastic modulus, curing shrinkage, and thermal stability to minimize localized stresses and structural failure arising from material mismatch and temperature fluctuations during package operation.

From the standpoint of thermomechanical reliability, CTE matching represents one of the most fundamental requirements for underfill materials. Owing to the mismatch in thermal expansion among the die, underfill, substrate, and interconnects, cyclic temperature variations generate repetitive thermomechanical stresses that are ultimately concentrated around solder joints. By reducing its own CTE and promoting more uniform deformation across the package, the underfill can alleviate the thermomechanical loading imposed on the interconnects and thereby mitigate solder-joint fatigue. In practice, high loadings of low-expansion inorganic fillers are commonly introduced to reduce the CTE of underfill materials to approximately 20–30 ppm/°C below the glass transition temperature (T_g_), while more advanced formulations can achieve even lower values. Further reductions can be obtained by tailoring particle size, optimizing filler morphology, and exploiting interfacial confinement effects, which restrict the thermally induced motion of polymer chains. Meanwhile, the continuous increase in power consumption of advanced logic devices has made thermal management an increasingly important function of underfills. Conventional epoxy resins possess predominantly amorphous molecular structures and lack long-range ordered pathways for heat transport, resulting in a relatively low thermal conductivity of approximately 0.2 W/(m·K), which is insufficient for applications involving high heat fluxes. Incorporation of highly thermally conductive fillers such as AlN, BN, and SiC has therefore been widely explored to establish interconnected heat-transfer pathways and enhance thermal spreading within the underfill. Recent research-grade thermally conductive underfill composites have achieved thermal conductivities above 1 W/(m·K), while highly filled AlN/BN hybrid systems in molded underfills have in some cases exceeded 10 W/(m·K) [8]. Such high thermal conductivity, however, is generally achieved at the expense of processability, because highly conductive fillers are often relatively rigid and possess large specific surface areas. Their incorporation at high concentrations can sharply increase viscosity and narrow the processing window, which is particularly challenging for conventional capillary underfill processes. The design of thermally conductive underfills must therefore reconcile efficient heat transport with manufacturing compatibility.

Beyond thermal characteristics, rheological behavior is a critical determinant of whether an underfill can satisfy industrial processing requirements. In CUF processing, the resin must rapidly penetrate and completely occupy the narrow gap, typically tens of micrometers wide, between the chip and substrate. The filling dynamics are governed collectively by viscosity, surface tension, and interfacial wettability. According to capillary-flow principles, reducing viscosity generally accelerates filling; consequently, conventional capillary underfills require sufficiently low viscosity under the relevant dispensing temperature and shear-rate conditions to enable rapid filling of narrow gaps. However, both CTE reduction and thermal-conductivity enhancement generally require substantial incorporation of inorganic fillers. As the filler volume fraction increases, stronger particle–particle friction and network formation can cause a pronounced increase in viscosity. This inherent conflict between high filler loading and low-viscosity processing constitutes one of the defining challenges that distinguishes underfill materials from conventional polymer composites. Current approaches for alleviating this trade-off include the use of spherical fillers, multimodal particle-size distributions, surface coupling modification, and interfacial lubrication strategies.

Underfills also serve a dual mechanical function within the package by constraining interconnect deformation while accommodating thermally induced stress. Their mechanical properties must therefore be carefully balanced. An excessively low elastic modulus may provide insufficient constraint against the plastic deformation of solder joints during thermal cycling, whereas an excessively high modulus can intensify the transfer of thermomechanical stresses toward the silicon die and low-k dielectric layers, thereby increasing the susceptibility to cracking and delamination. Advanced underfill systems consequently require an appropriate modulus range that provides sufficient structural reinforcement without excessively restricting stress relaxation. Meanwhile, although the highly crosslinked structure of epoxy resins provides excellent stiffness and thermal resistance, it also gives rise to intrinsic brittleness. During prolonged thermal cycling, cracks preferentially initiate at filler–matrix interfaces or other regions of localized stress concentration, making fracture-toughness enhancement and interfacial strengthening important components of mechanical optimization. Conventional toughening approaches, however, often involve a concomitant reduction in modulus or T_g_. Achieving a simultaneous combination of high toughness, high modulus, and high thermal resistance therefore remains a significant challenge for the design of advanced underfill materials.

Reliability ultimately determines the practical viability of underfill materials in advanced packaging. Devices and packages are expected to withstand prolonged exposure to thermal fluctuations, humid environments, and mechanical loading; accordingly, an underfill must not only preserve its own structural integrity but also maintain robust interfaces with the die, solder joints, and substrate throughout service. Among the various reliability-related factors, moisture uptake is particularly important for epoxy-based underfills. The polar functionalities within epoxy networks facilitate water ingress, which can induce polymer plasticization and hygroscopic swelling, thereby altering the glass transition temperature (T_g_), dimensional stability, and interfacial integrity [9]. Highly reliable underfill formulations are therefore typically required to withstand damp-heat qualification protocols, such as the 85 °C/85% relative humidity (85/85) test. In addition, the cyclic thermomechanical stresses generated during temperature excursions can drive solder-joint fatigue, interfacial delamination, and crack propagation, ultimately governing package lifetime. With the rapid adoption of 5G millimeter-wave technologies and the prospective development of 6G communication systems, underfill materials are also facing increasingly stringent dielectric requirements, particularly low dielectric constant and low dielectric loss, to minimize signal attenuation during high-frequency transmission.

Taken together, the development of epoxy-based underfills is moving beyond conventional single-property reliability enhancement toward multifunctional and synergistic materials design. The relevant performance metrics are strongly interdependent and often involve inherent trade-offs. Increasing filler loading can simultaneously reduce the CTE and facilitate heat dissipation but generally compromises flowability and processability. Increasing crosslink density improves thermal and dimensional stability, yet may come at the expense of fracture toughness. Likewise, strengthening interfacial adhesion can enhance package reliability, while excessive interfacial confinement or the formation of interfacial layers may alter thermal-transport pathways. Consequently, the next generation of high-performance underfills should not be designed simply by maximizing individual property metrics. Instead, a more effective strategy is to establish coordinated control across resin molecular architecture, filler engineering, interfacial chemistry, and processing conditions, thereby achieving a balanced combination of thermal, rheological, mechanical, and reliability performance.

## 3. Progress in the Regulation of Thermal Properties

Thermal properties are among the key determinants of the long-term reliability of underfill materials and are primarily associated with thermal-expansion compatibility and heat-transport capability. On the one hand, the underfill must mitigate the thermal-expansion mismatch among the die, solder joints, and substrate to prevent excessive interfacial stresses during thermal cycling. On the other hand, the increasing power density of advanced chips and the evolution of advanced packaging architectures demand more efficient heat dissipation from underfill materials. Accordingly, the thermal-property engineering of underfill materials is evolving from conventional single-filler modification toward multiscale synergistic optimization of filler architecture, interfacial interactions, and thermo-mechanical properties.

### 3.1. Strategies for Low-CTE Matching

Designing underfills with a low coefficient of thermal expansion (CTE) is fundamental to improving the thermomechanical reliability of electronic packages. Unfilled epoxy resins typically exhibit a CTE of approximately 60–80 ppm/°C below the T_g_, which differs substantially from that of silicon. In industrial underfills, high loadings of low-CTE inorganic fillers are therefore commonly employed to reduce the composite CTE to approximately 20–30 ppm/°C, thereby alleviating thermal-expansion mismatch with the die, solder joints, and organic substrate. Among these fillers, spherical SiO_2_ is the most extensively used because of its exceptionally low CTE (~0.5 ppm/°C), excellent electrical insulation, and well-established manufacturing technology.

Early studies primarily focused on establishing the relationship between filler loading and the CTE of epoxy composites. Wong et al. [10] compared the thermal conductivity, elastic modulus, and CTE of epoxy composites containing different ceramic fillers and examined the influence of filler loading on their thermomechanical properties. Although increasing the filler fraction is an effective means of lowering CTE, it simultaneously increases viscosity and compromises flowability, thereby narrowing the processing window of underfills. Lee et al. [11] compared SiO_2_-, Al_2_O_3_-, BN-, and diamond-filled systems and found that high-aspect-ratio BN was more effective in reducing CTE but caused a pronounced increase in viscosity. By contrast, spherical SiO_2_ provided a more favorable balance between thermomechanical performance and processability. Tang et al. [12] further investigated the effect of SiO_2_ loading on the fatigue behavior of underfill materials and demonstrated that filler content has a substantial influence on fatigue reliability. Collectively, these studies indicate that CTE reduction cannot be achieved simply by maximizing filler loading; instead, the structural characteristics of the fillers and the state of the filler–matrix interface must also be taken into account.

Tailoring the particle-size distribution represents an important strategy for improving filler packing efficiency. A monomodal particle-size distribution generally limits the achievable filler loading because of inefficient packing, whereas multimodal particle-size distributions can increase packing density and reduce resin-rich regions, thereby improving the thermomechanical characteristics of the composite. Guo et al. [13] employed a sol–gel process to construct a bimodal SiO_2_ filler system consisting of submicrometer- and nanoscale particles. The larger particles effectively mitigated nanoparticle agglomeration, allowing the formulation to achieve reduced viscosity while simultaneously improving T_g_ and thermal stability. Chen et al. [14] subsequently applied a bimodal particle-size design to a spherical Al_2_O_3_/epoxy underfill system. By blending 30 and 5 μm spherical Al_2_O_3_ particles at a volume ratio of 80:20 and a total filler loading of 50 vol%, the composite exhibited a shear viscosity of 21.8 Pa·s (25 °C, 1.0 s^−1^) and a thermal conductivity of 1.364 W/(m·K). This formulation represents a favorable trade-off between flowability and thermal performance enabled by bimodal particle packing. These findings highlight the potential of multiscale particle packing to improve the utilization efficiency of inorganic fillers through deliberate control of their spatial arrangement.

Beyond particle-size engineering, tailoring filler morphology and internal architecture offers another route toward CTE reduction. Conventional spherical SiO_2_ primarily suppresses polymer-chain mobility by reducing the free volume of the polymer matrix, whereas structurally engineered fillers can introduce additional nanoscale confinement. Zhao et al. [15] replaced conventional nonporous SiO_2_ with mesoporous SiO_2_ particles (MSPs). The internal mesoporous architecture facilitates the penetration of epoxy molecules into the pores and imposes additional confinement on polymer-chain motion after curing. As a result, MSP-filled composites exhibited a stronger reduction in CTE at comparable filler loadings while retaining favorable fracture toughness. Further investigation revealed that MSP particle size strongly affects interfacial interactions and thermomechanical behavior. Particles with an intermediate size provided a more favorable balance among dispersion, interfacial confinement, and stress relaxation, demonstrating that filler architecture can influence CTE regulation beyond the effect of filler loading alone. Nevertheless, the porous structure of MSPs may introduce concerns regarding moisture absorption and long-term hygrothermal reliability, which should be carefully considered in practical applications.

Beyond optimizing the intrinsic structure of fillers, engineering the filler–matrix interface has emerged as an effective approach for further suppressing the CTE. Because polymer-chain mobility is restricted within the interfacial region, a mechanically constrained interphase with reduced thermal expansion can develop around the filler. Such an interphase can increase the effectiveness of CTE suppression without requiring a further increase in filler loading. Li et al. [16] investigated the interfacial effects of SiO_2_ modified with different silane coupling agents and demonstrated that the chemical functionality introduced at the filler surface has a pronounced influence on CTE reduction. In particular, surfaces bearing nonpolar functional groups exhibited a greater ability to suppress thermal expansion. Lee et al. [17] employed epoxy-functional silane pretreatment to improve the dispersion of SiO_2_ within the epoxy matrix, reducing the CTE from 42.6 to 29.1 ppm/°C while simultaneously enhancing the shear strength after thermocompression bonding. Polymer-based interfacial layers have also attracted increasing interest because their abundant functional groups provide greater flexibility for tailoring interfacial interactions. Guo et al. [18] surface-functionalized SiO_2_ nanoparticles with branched polyethyleneimine (PEI). The modification not only improved particle dispersion but also substantially reduced the viscosity of the formulation. At an identical SiO_2_ loading, the CTE was further reduced by 4.8 ppm/°C, corresponding to an effect comparable to increasing the nanoscale SiO_2_ content by approximately 10 wt%. This result highlights the potential of molecular-level interfacial design to improve filler utilization efficiency and thereby alleviate the inherent trade-off between low CTE and high viscosity.

Meanwhile, multifunctional filler architectures are emerging in response to the thermal-management requirements of high-power advanced packaging. Zhao et al. [19] developed a binary SiO_2_/SiC filler system that integrates the low-CTE characteristics of SiO_2_ with the high thermal conductivity of SiC. The resulting composite maintained a CTE of approximately 23 ppm/°C while exhibiting enhanced thermal conductivity. By incorporating low-expansion and heat-transfer functions within a unified filler architecture, this strategy provides a viable route toward multifunctional underfills in which thermomechanical and thermal-management requirements can be addressed simultaneously.

Overall, the CTE engineering of underfill materials has evolved from the straightforward approach of increasing filler loading toward a more sophisticated combination of particle-size optimization, architectured fillers, and interfacial chemical design. Future development of low-CTE underfills will increasingly hinge on improving filler utilization efficiency—while preserving processability and long-term reliability—rather than pursuing CTE reduction solely through higher filler concentrations.

### 3.2. Thermal-Conductivity Enhancement and Interfacial Thermal Resistance Regulation

The rapid growth of high-performance computing, artificial intelligence processors, and three-dimensional heterogeneous integration has continuously increased chip power density, making thermal management an increasingly important factor governing the reliability of advanced packages. From the perspective of heat-transfer mechanisms, epoxy resins lack intrinsically continuous and efficient pathways for thermal transport, and heat is predominantly transferred through relatively inefficient energy propagation within their disordered molecular networks. Accordingly, establishing interconnected heat-conduction pathways while reducing interfacial thermal resistance (ITR) constitutes a fundamental strategy for enhancing the thermal conductivity of epoxy-based underfills, typically through the incorporation of highly thermally conductive fillers. Unlike conventional thermal-management composites, however, underfills must simultaneously accommodate microscale capillary filling, low-viscosity processing, electrical insulation, and thermomechanical reliability. Thermal-conductivity enhancement must therefore be pursued under multiple and often competing performance constraints.

**Figure 2:**
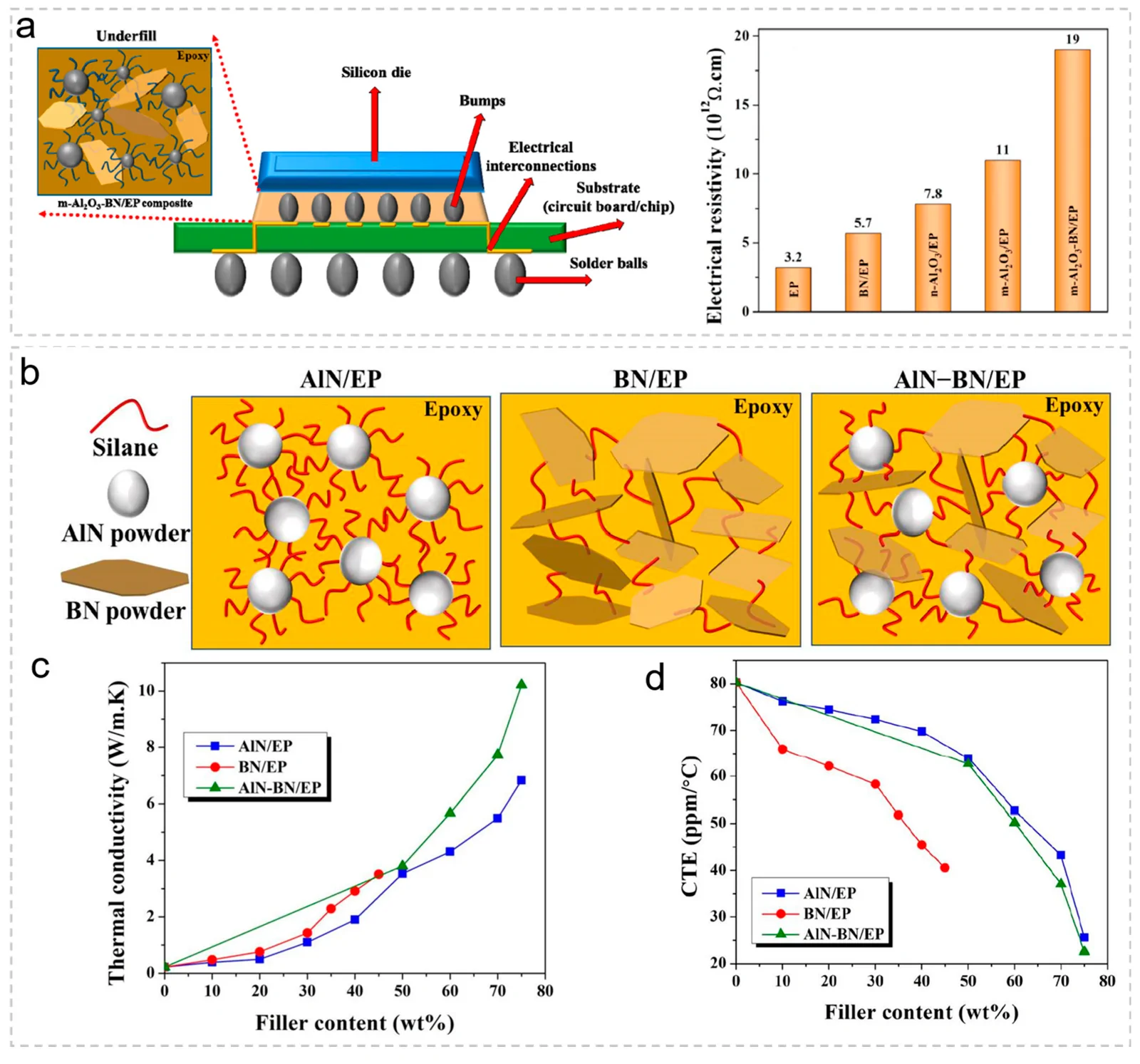
(**a**) Schematic of flip-chip underfill packaging and volume resistivity of epoxy resin composites at their maximum filler loadings [20]. (**b**) Schematic of the particle arrangement in the epoxy matrix. (**c**) TC of EP composites versus filler loading. (**d**) CTE below T_g_ graphs for EP composites versus filler loading [8].

Wen et al. [3] noted in their review in Advanced Materials that thermally conductive underfills should ideally combine a thermal conductivity above 1.0 W/(m·K), relatively low viscosity, and an appropriate CTE in the range of 25–30 ppm/°C. These criteria provide useful benchmarks for conventional advanced packaging; however, with the emergence of chiplet architectures and increasingly heterogeneous integration, stricter thermomechanical requirements, particularly lower CTE and improved reliability under complex thermal cycling conditions, may become necessary. Accordingly, research on thermally conductive underfills has gradually shifted from simply increasing filler loading toward filler-architecture engineering, multiscale network construction, and interfacial thermal-resistance regulation. Conventional ceramic fillers remain the dominant class of thermally conductive additives because of their electrical insulating characteristics and compatibility with electronic packaging processes. Among them, Al_2_O_3_ has been extensively employed because of its relatively low cost and good chemical stability. Its moderate intrinsic thermal conductivity, however, limits the achievable thermal performance of single-component Al_2_O_3_ systems in high-power packaging. Chen et al. [14] therefore enhanced heat-transfer efficiency through particle-architecture optimization and multicomponent filler blending. The incorporation of highly conductive two-dimensional fillers can further facilitate heat transport between conventional ceramic particles. Sanchez et al. [20] constructed a hybrid filler architecture consisting of BN nanosheets and microscale spherical Al_2_O_3_ particles. At a total filler loading of 80 wt%, the resulting composite exhibited a thermal conductivity of 1.72 W/(m·K) while retaining good electrical insulation (Figure 2a). The same research group subsequently replaced part of the Al_2_O_3_ with AlN and combined it with BN to establish a more efficient thermally conductive network [8] (Figure 2b). A thermal conductivity of 10.18 W/(m·K) was achieved at a hybrid filler loading of 75 wt%, accompanied by a reduction in CTE to 22.56 ppm/°C (Figure 2c,d). These results demonstrate that spatial and dimensional complementarity among ceramic fillers can substantially overcome the thermal-transport limitations of single-filler systems.

Beyond conventional ceramic fillers, carbon-based nanomaterials offer an alternative route toward thermal-conductivity enhancement at relatively low filler loadings owing to their intrinsically high thermal conductivity and favorable structural characteristics. Their application in epoxy matrices, however, is complicated by severe agglomeration, inadequate interfacial adhesion, and the loss of electrical insulation associated with highly conductive carbon networks. Consequently, recent efforts have increasingly focused on functionalizing carbon-based fillers to decouple their thermal and electrical characteristics. Cui et al. [21] employed a sol–gel process to deposit a SiO_2_ coating onto multiwalled carbon nanotubes (MWCNTs), producing an MWCNT@SiO_2_ hybrid structure. The insulating SiO_2_ shell improved nanotube dispersion and alleviated interfacial incompatibility between the filler and epoxy matrix. As a result, the MWCNT@SiO_2_ hybrid filler enabled an improvement in thermal conductivity at relatively low filler loadings of 0.5 and 1 wt%, while maintaining relatively high electrical resistivity. Hsiao et al. [22] further reported TRGO–SiO_2_ sandwich-structured nanosheets, in which the SiO_2_ layers suppressed the formation of electrically conductive networks while facilitating phonon transport. At a loading of only 1 wt%, the thermal conductivity increased from 0.20 W/(m·K) for neat epoxy to 0.322 W/(m·K). To address the excessive electrical conductivity of graphene, which limits its direct application in electronic encapsulation, insulating ceramic functionalization has also been explored. Sun et al. [23] modified graphene nanoplatelets with Al_2_O_3_ to construct Al_2_O_3_@GNP hybrid fillers. The epoxy composite containing 12 wt% of the hybrid filler achieved a thermal conductivity of 1.49 W/(m·K), corresponding to a 677% increase over neat epoxy, while retaining favorable electrical insulation. Du et al. [24] introduced an insulating MgO shell onto multiwalled carbon nanotubes (MgO@MWNTs), thereby improving their dispersion and interfacial adhesion within the epoxy matrix. The resulting composites exhibited substantially enhanced thermal conductivity while maintaining electrical insulation; at a filler loading of 2.0 wt%, the thermal conductivity increased by 89% relative to that of neat epoxy. These studies demonstrate that engineering an electrically insulating inorganic shell around highly conductive carbon fillers provides an effective means of reconciling high thermal conductivity with the electrical insulation required for electronic packaging.

Metallic nanostructures have also attracted increasing attention as thermally conductive fillers because of their high intrinsic thermal transport capability. Their application in epoxy underfills, however, is challenged by pronounced interfacial vibrational mismatch and the inherent electrical conductivity of metallic phases. Core–shell architectures have therefore emerged as a promising strategy for simultaneously addressing these limitations. Jiang et al. [25] investigated the influence of interfacial elastic-modulus mismatch on heat transport in TiO_2_-coated Ag nanowires. Chen et al. [26] subsequently developed SiO_2_-coated Ag nanowires (AgNWs@SiO_2_), in which the SiO_2_ layer served as an interfacial transition region to reduce the mismatch between the metallic filler and epoxy matrix. At a filler content of 4 vol%, the thermal conductivity increased from 0.19 W/(m·K) for neat epoxy to 1.03 W/(m·K), substantially exceeding that of the corresponding composite containing uncoated AgNWs (0.57 W/(m·K)). This finding indicates that appropriate interfacial-layer engineering can simultaneously improve filler dispersion and facilitate heat transfer across the metal–polymer interface. In addition to nanowire-based architectures, metallic core–shell particles provide another route toward electrically insulating, thermally conductive fillers. Mao et al. [27] reported Al@Al_2_O_3_ core–shell particles, in which the metallic Al core served as an efficient heat-transfer phase while the Al_2_O_3_ shell provided electrical insulation. At a filler loading of 60%, the resulting composite reached a thermal conductivity of 0.92 W/(m·K), approximately 4.2 times that of the neat epoxy. This architecture broadens the design space beyond conventional electrically insulating ceramics and provides an alternative strategy for developing thermally conductive underfills. Compared with single-filler formulations, multiscale hybrid architectures can generate more continuous heat-transfer pathways at reduced overall filler loadings. Hu et al. [28] employed high-aspect-ratio nanoscale fillers to bridge microscale ceramic particles, thereby constructing a hierarchical micro–nano thermally conductive network with substantially enhanced heat-transfer efficiency. Such results highlight the importance of spatial complementarity among fillers with different dimensions and morphologies in constructing efficient thermal-transport pathways within highly filled epoxy systems.

In addition to the intrinsic thermal conductivity of the fillers, interfacial thermal resistance (ITR) represents another major bottleneck to further improvement of the effective thermal conductivity. The vibrational spectra and phonon characteristics of inorganic fillers differ substantially from those of the epoxy matrix, leading to phonon scattering and energy dissipation as heat crosses the filler–matrix interface. Despite its importance, systematic investigation of ITR in underfill systems remains relatively limited. Existing interfacial-engineering studies have predominantly focused on particle dispersion, CTE matching, and mechanical reinforcement. For example, silane coupling agents, polymeric interfacial layers, and SiO_2_-coated architectures can improve filler–matrix compatibility, but their effects on interfacial vibrational coupling and phonon transport have yet to be adequately elucidated.

Future advances in thermally conductive underfills will therefore require a transition from macroscopic filler optimization toward molecular- and interface-level regulation. Combining molecular dynamics simulations with experimental characterization could help establish quantitative relationships among interfacial chemical structure, vibrational/phonon coupling, and thermal transport, thereby enabling rational design of low-ITR interfaces. Overall, the development of high-performance thermally conductive underfills requires simultaneous consideration of thermal conductivity, CTE, viscosity, electrical insulation, and long-term reliability rather than maximizing thermal conductivity alone. Achieving this multidimensional balance will be essential for meeting the increasingly stringent thermal-management requirements of advanced packaging.

## 4. Progress in the Regulation of Rheological Properties

Whereas thermal properties largely determine the service reliability of underfill materials, rheological properties govern their ability to be processed and successfully integrated into a package; together, these characteristics define the fundamental requirements for practical application. As advanced packaging progresses toward higher integration density, finer interconnect pitches, and larger die dimensions, CUF processes impose increasingly stringent demands on flowability. Underfill materials must rapidly penetrate gaps of only several tens of micrometers, or even narrower, between the die and substrate while also maintaining adequate storage stability and a well-controlled curing response. As discussed above, achieving low CTE and high thermal conductivity generally requires high loadings of inorganic fillers, which inevitably cause a sharp increase in viscosity and severely impede capillary flow. Accordingly, rheological optimization should not be viewed simply as a viscosity-reduction problem. Rather, the central challenge is to reconcile processability, dispersion stability, and curing behavior while retaining the thermal performance required for advanced packaging.

From a multiscale perspective, the rheological response of underfill materials is governed by three interconnected levels. The molecular structure of the continuous phase establishes the lower limit of viscosity; the microstructure of the concentrated particle suspension determines flow resistance at high filler loadings; and capillary transport under realistic packaging conditions ultimately dictates the efficiency of gap filling. Recent research has consequently expanded from empirical formulation optimization toward multiscale design strategies encompassing resin molecular engineering, filler engineering, interfacial chemistry, and capillary-flow dynamics.

### 4.1. Molecular Design of the Resin Matrix and Curing System

Underfills are typically highly filled epoxy composites whose rheological behavior is initially governed by the molecular characteristics of the continuous phase and the associated curing reactions. Coordinated molecular design of the resin matrix, curing system, and reactive diluents therefore provides a fundamental basis for reducing the viscosity of the continuous phase, widening the processing window, and simultaneously maintaining the desired thermomechanical properties after curing.

Commercial epoxy systems commonly employ diglycidyl ether of bisphenol A (DGEBA) and diglycidyl ether of bisphenol F (DGEBF) as the principal matrix resins. Although both are difunctional glycidyl ether epoxies, differences in their molecular backbones and epoxy equivalent weights result in substantially different viscosities. Previous studies have reported typical viscosities of approximately 2.5–4.5 Pa·s for DGEBF and 8.5–15 Pa·s for DGEBA at 25 °C. The lower viscosity of DGEBF, associated with its lower molecular weight and higher epoxy-group content per unit mass, facilitates filler wetting and dispersion and improves the flowability of highly filled formulations [29]. DGEBF is therefore frequently incorporated as a low-viscosity component to reduce the overall viscosity of epoxy formulations, whereas DGEBA contributes greater rigidity and favorable thermomechanical properties. In practical formulations, DGEBA/DGEBF blending is commonly adopted to lower the viscosity of the continuous phase while preserving the T_g_, modulus, and dimensional stability of the cured network.

Beyond the matrix resin, the molecular structure of the curing agent and the kinetics of the curing reaction critically determine the evolution of viscosity with temperature and time, while also governing the architecture of the resulting crosslinked network and its thermomechanical characteristics. Anhydride-, amine-, and imidazole-based curing systems exhibit distinct reactivities and network-forming mechanisms, allowing gelation time, crosslink density, final T_g_, and modulus to be tailored through curing-agent molecular design. Khalid et al. [30] compared the effects of anhydride crosslinkers with different molecular structures on the thermomechanical properties of epoxy underfills and found that the crosslinker structure substantially affected thermal stability, hardness, flexibility, and CTE. Systems incorporating aromatic structures exhibited lower CTE and higher hardness, whereas aliphatic systems provided greater flexibility. These findings demonstrate that curing agents are not merely reactive components controlling curing kinetics; their molecular structures also provide an important lever for coordinating rheological evolution with the thermomechanical properties of the cured underfill.

For one-component underfill formulations, latent curing agents are essential for reconciling storage stability at ambient temperature with efficient thermal curing. Conventional dicyandiamide (DICY) exhibits relatively low reactivity at room temperature while providing good storage stability, making it one of the widely used latent curing agents for one-component epoxy systems. However, DICY generally requires relatively high curing temperatures to achieve sufficient conversion. Accelerators are therefore commonly incorporated into practical formulations to enhance their reactivity, lower the curing temperature, and shorten the curing time. In recent years, research has increasingly moved beyond conventional DICY/accelerator combinations toward regulating curing activity through molecular engineering of curing agents, chemical blocking, and physical confinement. Imidazole-based accelerators are particularly attractive because of their high catalytic efficiency toward epoxy curing. Their relatively high activity at ambient temperature, however, can induce slow curing during storage and consequently increase formulation viscosity when directly incorporated into one-component systems. Developing latent imidazole accelerators that retain high catalytic activity upon heating while remaining sufficiently inactive at room temperature has therefore become an important research direction. Arimitsu et al. [31] functionalized imidazole at the nitrogen atom with a thermally labile, branched long-chain alkyl succinate group, prepared via a base-catalyzed aza-Michael addition of imidazole to bis(2-isopropyl-5-methylhexyl) fumarate. The resulting liquid adduct undergoes thermal decomposition at 200–280 °C via a retro-Michael addition, regenerating the free imidazole together with the fumarate diester. In the epoxy resin the decomposition shifts to a lower temperature (DTA exotherm ≈150 °C), and the released imidazole cures jER828 at 120–150 °C. The latent-agent/epoxy mixture, however, remains fluid at room temperature for more than 12 days. Jung et al. [32] developed an imidazole-based thermally responsive latent curing system using a Diels–Alder reaction. In this design, benzyl-2-methylimidazole serves as the active species, while a thermally responsive Diels–Alder network is constructed from a tetrafunctional furan and a bismaleimide. The resulting architecture simultaneously provides physical shielding through the dynamic network and chemical protection of the imidazole moieties, thereby suppressing their catalytic activity during storage. Upon heating, dissociation of the Diels–Alder network exposes the imidazole groups and activates epoxy curing. Compared with free imidazole, the resulting microspheres exhibited substantially improved storage stability when dispersed in the epoxy resin, maintaining stability for approximately 30 days at 20 °C.

Ozsaltik et al. [33] reported a thermally latent poly(2-oxazoline)-imidazole (POZ-Im) curing agent, in which imidazole groups were covalently incorporated at the chain ends of poly(2-ethyl-2-oxazoline), poly(2-propyl-2-oxazoline), and poly(2-phenyl-2-oxazoline) through a one-pot synthesis. Their results demonstrated that variations in the side-chain structure could substantially alter formulation compatibility, curing onset temperature, gelation time, and storage stability. Among the investigated formulations, several POZ-Im/DGEBA systems exhibited prolonged gelation times and favorable latency. These findings indicate that latent curing-agent design is evolving from conventional modulation of small-molecule reactivity toward molecular engineering based on polymer architecture and interfacial states. Rheological characterization is also becoming increasingly integrated into this process to directly assess gelation behavior and storage stability.

In contrast to chemical modification of the curing species, microencapsulation provides an alternative route to latency by physically isolating curing agents or accelerators from the epoxy matrix. This approach is particularly attractive from an application perspective because the curing components can remain effectively dormant during storage and be released upon thermal activation. Zhang et al. [34] prepared organic–inorganic hybrid microcapsules containing 2-phenylimidazole (2-PhIm) through Pickering emulsion polymerization and incorporated them into a DGEBA/anhydride system. The addition of 5 wt% microcapsules substantially extended the room-temperature working life of the one-component formulation. Upon heating, 2-PhIm was released from the capsules and promoted epoxy curing, while the microcapsules caused no significant deterioration in the thermomechanical properties of the cured material. This study demonstrates that microencapsulation can not only enhance storage stability but also provide additional control over the activation temperature of the accelerator through deliberate regulation of shell composition and architecture. Related microencapsulation strategies have also been extended to other imidazole-based curing accelerators. Ham et al. [35] employed polycaprolactone (PCL) as the shell material and encapsulated 2-phenylimidazole through a solvent-evaporation process for latent curing of epoxy systems. Xing et al. [36] further investigated shell materials such as polyetherimide (PEI), together with different emulsion and solvent-evaporation processes, to improve both the storage stability of the microcapsules and their thermally triggered curing performance.

In the formulation of active diluents, their primary function is to reduce the viscosity of the continuous phase, thereby facilitating filler wetting and dispersion as well as capillary flow. Conventional monofunctional glycidyl ether diluents offer very low viscosity and high dilution efficiency. However, because each molecule contains only one epoxy group, their incorporation into the curing network lowers the average functionality and crosslink density of the formulation, potentially compromising T_g_, modulus, and thermal resistance. For underfill systems, therefore, the design of reactive diluents should not be driven solely by minimizing viscosity; dilution efficiency must be considered in tandem with preservation of the structural integrity of the cured network.

More recently, reactive diluents have increasingly evolved toward bio-based and multifunctional molecular architectures. Chen et al. [37] synthesized poly(epoxidized cardanol glycidyl ether) (PECGE) from cardanol and introduced it into a petroleum-derived epoxy resin as a bio-based multifunctional reactive diluent. PECGE was found to reduce the viscosity of the epoxy formulation while simultaneously improving the tensile strength, elongation at break, and thermal resistance of the cured material, with only minor changes in flexural and compressive strength. PECGE can therefore serve as a multifunctional reactive component that combines viscosity reduction with mechanical-property retention or enhancement. Hyperbranched epoxy architectures provide another promising platform for reactive-diluent design. Owing to their highly branched molecular structures and numerous terminal epoxy groups, hyperbranched epoxies can, when their molecular weight and functionality are appropriately controlled, combine favorable flowability with high chemical reactivity. Moreover, the balance between rigidity and flexibility of the resulting crosslinked network can be tailored through molecular branching and segmental design. Previous studies have incorporated epoxy/hyperbranched polymer systems into liquid molding processes such as resin transfer molding and investigated the effects of reactive diluents on processing viscosity and curing behavior [38].

Overall, molecular design of the resin matrix, curing system, and reactive diluents has progressed from simply lowering the viscosity of the continuous phase toward coordinated regulation of molecular architecture, curing kinetics, and crosslinked-network structure. Optimization of the continuous phase alone, however, is insufficient to satisfy the processing requirements of highly filled underfills. Further reduction in viscosity and expansion of the flow-processing window will require coordinated control over filler particle-size distribution, particle morphology, surface modification, and polymer–filler interfacial interactions.

### 4.2. Effects of Filler Engineering and Interfacial Regulation on Rheological Behavior

Once the filler volume fraction exceeds approximately 40 vol%, an underfill formulation enters the regime of a concentrated suspension, in which its rheological response is increasingly governed by particle-packing characteristics and interparticle/interfacial interactions rather than by the properties of the continuous phase alone. At this stage, further optimization of the resin matrix has a limited effect on viscosity. More effective strategies involve increasing packing efficiency, weakening particle–particle interactions, and maintaining a well-dispersed filler population within the resin. Accordingly, filler engineering and interfacial chemistry have become central to rheological regulation in highly filled underfill systems.

Theories of concentrated-suspension rheology provide an important framework for designing highly filled underfill formulations. Compared with the Einstein model, which is primarily applicable to dilute suspensions, the Krieger–Dougherty (K–D) model [39] provides a more appropriate description of the viscosity–concentration relationship over moderate-to-high filler volume fractions. However, it assumes rigid, non-interacting spherical particles and therefore has limitations for describing interparticle interactions and nonspherical fillers commonly encountered in underfill formulations. According to the K–D model, as the filler volume fraction (φ) approaches the maximum packing fraction (φ_m_), the suspension viscosity increases nonlinearly and theoretically diverges as φ approaches φ_m_. Thus, when the properties of the continuous phase and the intrinsic characteristics of the particles are otherwise comparable, increasing φ_m_ can substantially reduce the viscosity at a given filler loading. Because φ_m_ is strongly dependent on particle-size distribution, particle morphology, and dispersion state, this framework provides a theoretical basis for improving packing efficiency through multimodal particle-size design and thereby reducing flow resistance in highly filled systems.

Building on the above framework, multimodal particle-size distribution has become one of the most established viscosity-reduction strategies for industrial underfills. By combining large, intermediate, and small particles within a hierarchical packing structure, the smaller particles can occupy the interstitial spaces between larger particles, increasing the overall packing density and thereby reducing suspension viscosity at a given total filler loading. Chen et al. [14] constructed a bimodal system using spherical Al_2_O_3_ particles with different particle sizes and demonstrated that a small-particle volume fraction of approximately 30% provided a favorable combination of high packing density and low flow resistance. This optimized packing configuration simultaneously improved the CTE and thermal conductivity while substantially enhancing processability. Compared with monomodal systems, multimodal particle-size distributions can maintain relatively favorable capillary filling behavior even at high filler loadings and have therefore been widely adopted in the formulation of high-performance underfills.

The viscosity-reduction benefits of multimodal packing extend beyond microscale fillers and have also been demonstrated for nanoscale systems. Guo et al. [13] synthesized SiO_2_ nanospheres with a bimodal particle-size distribution through a one-pot process and incorporated them into epoxy-based underfill composites. Compared with monodisperse nanoscale SiO_2_, the bimodal architecture improved particle dispersion within the resin matrix and substantially reduced the viscosity of the highly filled formulation, thereby enhancing its processing characteristics. Meanwhile, to further improve thermal transport, Feng et al. [40] designed a multiscale Al_2_O_3_/epoxy composite based on a close-packing model. At an identical total filler loading of 50 vol%, the multiscale filler architecture generated more effective heat-transfer pathways than the corresponding single-size system, resulting in higher thermal conductivity. Rheological characterization further confirmed that the synergistic particle-size effect contributed positively to the flow behavior of the formulation.

Beyond particle-size distribution, filler geometry also plays a decisive role in determining the rheological behavior of highly filled systems. Spherical particles generally exhibit relatively low hydrodynamic resistance and weak interparticle friction, allowing high filler loadings to be achieved without excessive viscosity. Consequently, fused spherical SiO_2_ remains the principal filler used in commercial underfill formulations. In contrast, high-aspect-ratio fillers such as platelet-like BN and fibrous materials are more effective in establishing thermally conductive networks, but their large effective hydrodynamic volume can substantially increase suspension viscosity. These fillers are therefore typically incorporated in limited amounts as secondary thermal-conductivity enhancers rather than replacing spherical particles as the dominant filler phase. Fumed SiO_2_, by comparison, has an extremely high specific surface area and can form a three-dimensional particle network through interparticle hydrogen bonding. This network imparts yield-stress and thixotropic characteristics to the formulation, which can be beneficial for suppressing filler sedimentation during storage. Excessive fumed SiO_2_, however, markedly increases the low-shear viscosity and consequently impairs capillary filling, making precise control of its loading essential.

As the filler content increases further, stronger van der Waals interactions and hydrogen bonding between particles promote agglomeration, which becomes a major contributor to the rapid increase in viscosity. Therefore, beyond optimizing filler size, morphology, and spatial packing, regulating interparticle interactions through interfacial chemistry to suppress agglomeration and improve filler dispersion has emerged as an important strategy for controlling the rheological behavior of highly filled underfill systems. Among various approaches, silane coupling agents remain one of the most widely used methods for filler surface modification. Li et al. [16,41] systematically investigated the effects of silica surface modification with silanes bearing different terminal functional groups on the rheological, thermomechanical, and adhesion properties of epoxy underfill materials, establishing a structure–property relationship between filler surface chemistry and macroscopic performance. The results showed that modification with relatively nonpolar groups, such as methacryloxy and phenyl groups, effectively reduced the system viscosity, with methacryloxy-functional silane exhibiting the most pronounced viscosity-reducing effect. In contrast, amino-functional silanes underwent stronger chemical interactions with the epoxy matrix and promoted the formation of a three-dimensional network structure in the interfacial region, thereby resulting in higher viscosity. Notably, phenyl-silane modification not only reduced viscosity but also substantially lowered the coefficient of CTE of the composites. In addition, modification with nonpolar groups significantly enhanced the adhesion strength of the underfill materials and shifted the failure mode from interfacial adhesive failure to cohesive failure. These findings demonstrate that filler surface chemistry governs not only the flow behavior of highly filled systems but also filler dispersion, interfacial adhesion, and thermomechanical response, highlighting the importance of balancing interparticle interactions and matrix–filler interfacial interactions in the design of high-performance underfill materials.

Beyond small-molecule coupling agents, polymer interfacial layers have emerged as an important direction in the surface engineering of highly filled composite systems, owing to their richer molecular architectures and greater tunability of interfacial chemistry. Roh et al. [42] employed plasma polymerization to construct polymer coatings bearing amino, hydroxyl, or thiol groups on the surface of SiO_2_ particles. They found that the polymer coatings not only modified the surface chemistry of SiO_2_ but could also participate in the epoxy curing reaction, thereby strengthening the interfacial chemical bonding between the fillers and the resin matrix. Further studies on polystyrene-grafted SiO_2_ nanoparticles demonstrated that the interpenetration between grafted chains and the matrix, as well as the grafting parameters, can substantially affect the viscoelastic behavior and viscosity of the composite systems [43]. Guo et al. [18] further constructed a flexible PEI interfacial layer on SiO_2_ particles through electrostatic self-assembly, enabling uniform surface coverage. The flexible PEI chains provide steric hindrance that reduces direct particle–particle contact and interparticle friction, thereby lowering the system viscosity by 79.7% while improving storage stability. Meanwhile, the amino groups of PEI can participate in the epoxy curing reaction, strengthening the interfacial chemical bonding between the fillers and the matrix and thereby achieving a synergistic improvement in viscosity reduction, dispersion stability, and interfacial adhesion. Compared with conventional small-molecule coupling agents, polymer interfacial layers introduce multiple interfacial regulation mechanisms, including steric stabilization, polymer-chain interpenetration, and interfacial chemical reactions, thereby providing greater design flexibility for the simultaneous optimization of rheological and interfacial properties in highly filled underfill materials.

**Figure 3:**
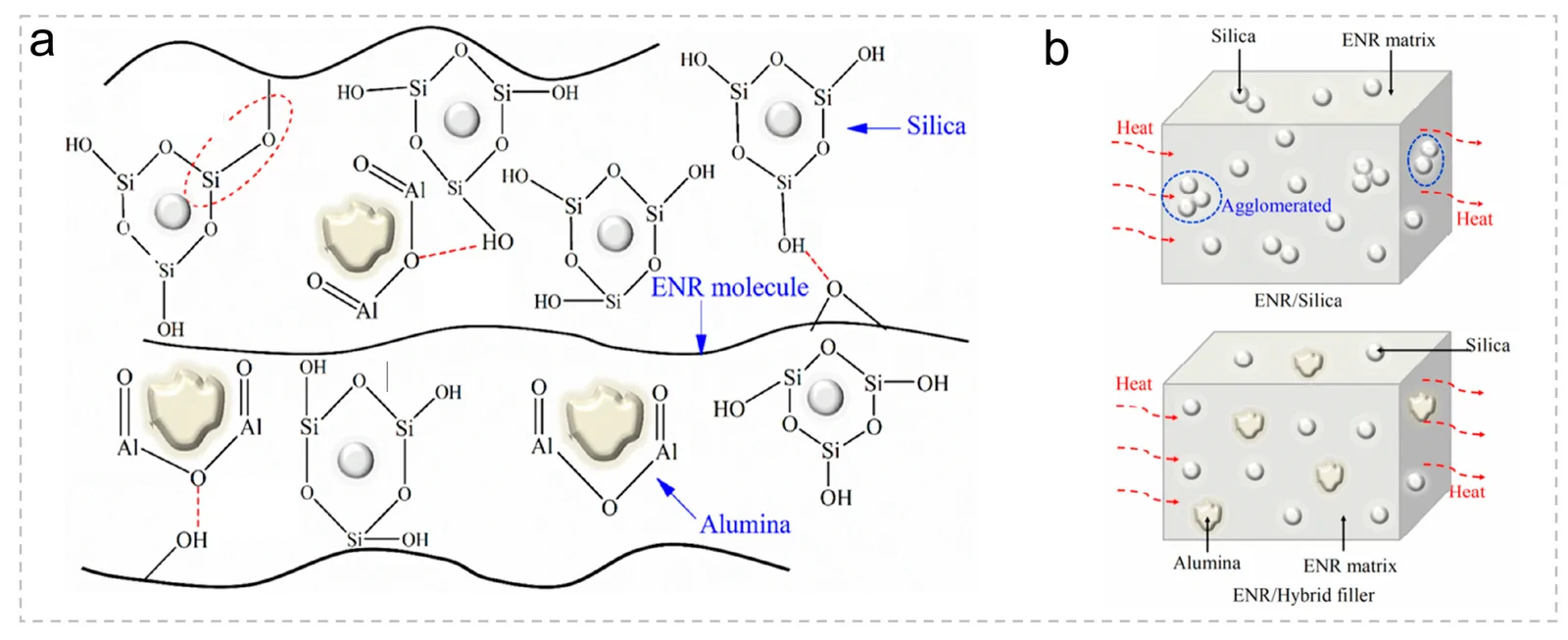
(**a**) Schematic illustration of the possible chemical reactions and interfacial interactions between ENR and the hybrid fillers, including rubber–filler interactions and hydrogen bonding between alumina and silica, ENR and silica, and ENR and alumina. (**b**) Schematic illustration of thermally conductive pathways formed by the hybrid fillers in the ENR matrix [44].

Notably, interfacial regulation is not limited to the pretreatment of filler surfaces. Tailoring the molecular structure of the matrix to strengthen matrix–filler interactions can likewise improve dispersion stability in highly filled systems. Yangthong et al. [44] demonstrated that, in an epoxidized natural rubber (ENR) system containing 50 mol% epoxy groups, hydrogen bonding between surface silanol groups on SiO_2_ and epoxy groups in the matrix (Figure 3a) enhances matrix–filler interactions, thereby enabling the uniform co-dispersion of hybrid Al_2_O_3_/SiO_2_ fillers without surface treatment. At the same filler loading, the hybrid system exhibited a lower viscosity than the SiO_2_-filled system, while its thermal conductivity was further enhanced (Figure 3b), indicating that molecular matching between matrix polarity and filler surface chemistry can not only promote particle dispersion but also simultaneously improve processability and thermal transport. This finding provides useful guidance for epoxy underfill systems: by tailoring the molecular structure of the matrix and its interfacial interactions with fillers, in conjunction with moderate surface modification, dispersion stability, processability, and thermomechanical performance can potentially be balanced at high filler loadings.

Overall, rheological regulation of highly filled underfill systems has evolved from relying primarily on particle-size optimization toward the integrated engineering of filler architecture and interfacial chemistry. Multimodal particle-size distributions improve packing efficiency, spherical fillers minimize flow resistance, and interfacial modification further weakens interparticle interactions. Through the combined implementation of these strategies, highly filled formulations can retain favorable processability while delivering the thermal performance required for advanced packaging. Nevertheless, existing studies have focused predominantly on optimizing steady-state viscosity, whereas the thixotropic recovery behavior, long-term storage stability, and particle-network restructuring under complex shear histories remain insufficiently understood. These dynamic rheological characteristics can ultimately have a direct impact on the actual capillary-filling behavior of underfill materials and therefore warrant greater attention in future studies.

### 4.3. Capillary Flow Behavior and Processability

For CUF processing, viscosity is only one of the factors governing filling efficiency. The actual transport of underfill through the microscale gap between the die and substrate is simultaneously influenced by wettability, gap dimensions, resistance imposed by the solder-ball array, and curing kinetics. Accordingly, evaluation of underfill rheology should extend beyond conventional steady-state rheological parameters and incorporate capillary-flow behavior under representative packaging conditions.

The classical Washburn equation establishes a quantitative relationship between capillary filling distance and key parameters, including liquid viscosity, surface tension, contact angle, and gap dimensions, providing a fundamental framework for analyzing underfill flow behavior [45]. Within this framework, reducing viscosity, improving wettability, and increasing gap height are generally expected to shorten the filling time. However, the Washburn model is based on the assumption of Newtonian flow and therefore cannot fully capture the actual transport behavior of highly filled underfill formulations. As filler loading increases, the formulation increasingly exhibits non-Newtonian characteristics, with flow behavior being affected by particle rearrangement, shear thinning, and local flow resistance imposed by the solder-ball array. Consequently, the applicability of the classical model becomes increasingly limited under advanced packaging conditions.

To address these limitations, Han, Wang, and co-workers [46] conducted systematic analyses of capillary flow during flip-chip underfilling and identified dynamic changes in contact angle, localized flow resistance induced by the solder-ball array, and non-Newtonian rheology as critical factors governing filling efficiency. Wan et al. [47] subsequently introduced a power-law fluid model into the analysis of underfill flow and developed an analytical description applicable to non-Newtonian formulations. Combined with experimental validation, the model provided more accurate predictions of underfill advancement and filling time than the conventional Washburn model [48]. These studies have progressively shifted the theoretical description of underfill transport from classical capillary-flow models toward non-Newtonian frameworks that better represent realistic processing conditions, providing an important basis for optimizing CUF processes in advanced packaging.

Beyond the intrinsic rheological characteristics of the fluid, interfacial wetting behavior is another critical determinant of the processability of underfill materials. Wang et al. [49] systematically investigated the viscosity, surface tension, and contact angle of underfill formulations and demonstrated that rapid filling requires not only low viscosity but also a sufficiently low contact angle at the processing temperature. Meanwhile, the viscosity of the underfill increases progressively during curing, rendering capillary filling inherently time-dependent. Their results further showed that, even for the same underfill formulation, differences in substrate surface energy can lead to markedly different wetting behavior, with higher-energy substrates exhibiting more favorable wetting and filleting characteristics. Stencel et al. [50] employed a two-phase-flow numerical model based on the Volume of Fluid (VOF) method to simulate capillary underfilling and experimentally validated both the flow-front evolution and filling velocity. The results demonstrated that the non-Newtonian viscosity characteristics at low shear rates and the time-dependent contact angle substantially affect the accuracy of flow prediction, with discrepancies between the experimental and simulated results limited to approximately 1.48–3.34%. These findings indicate that the processability of underfill materials cannot be determined by viscosity alone; rather, it arises from the coupled effects of viscosity, surface tension, contact angle, and their evolution with temperature and time. For substrates such as bismaleimide triazine (BT), FR-4 glass-epoxy laminate, ceramics, and flexible polyimide (PI), which differ considerably in surface chemistry and surface energy, underfill formulations must therefore be designed to balance viscosity reduction with interfacial wetting and compatibility. Processing parameters, including substrate preheating temperature and filling time, should likewise be optimized in conjunction with these interfacial characteristics.

It is also important to recognize that progressively narrower package gaps introduce several nonideal flow phenomena that are not adequately captured by conventional models. Shear-thinning behavior, for example, can reduce the local apparent viscosity near the advancing flow front and thereby facilitate filling [48]. When the die gap approaches the characteristic size of the filler particles, wall slip, particle filtration, and localized clogging can become increasingly important, potentially compromising filling uniformity. Consequently, practical formulations generally require the maximum filler particle size to be substantially smaller than the package gap, typically below approximately one-third and, for demanding applications, even one-fifth of the gap height, to maintain stable capillary transport. In addition, curing shrinkage after underfilling can have a significant influence on package reliability. Before gelation, the formulation retains sufficient mobility for shrinkage-induced stresses to be partially relaxed through molecular rearrangement. Once the gel point is reached, however, the material progressively loses its ability to flow, causing shrinkage-induced stresses to accumulate within the die–underfill–substrate assembly and potentially contributing to interfacial debonding and package warpage. Bao et al. [51] combined fiber Bragg grating (FBG) sensors, digital image correlation (DIC), and finite-element simulations to monitor the curing-shrinkage process of underfills in situ. Their results revealed the coupled relationship among curing kinetics, shrinkage strain, and package warpage, providing experimental insight for optimizing curing processes.

Overall, the processability of underfill materials depends not simply on achieving low viscosity but on the coordinated regulation of rheological behavior, interfacial wetting, and curing kinetics. As advanced packaging continues to evolve, conventional empirical formulation optimization is becoming increasingly inadequate. Future research should integrate multiscale flow simulations, in situ rheological characterization, and artificial-intelligence-assisted formulation design to establish quantitative relationships among material composition, microstructure, and capillary-flow behavior, thereby advancing underfill development from empirical optimization toward performance-oriented materials design.

## 5. Progress in the Regulation of Mechanical Properties

The preceding sections have addressed the service capability and processability of underfill materials from the perspectives of thermal and rheological properties, respectively. Once the underfill has completely filled the interfacial gap and undergone crosslinking, however, the focus of performance evaluation shifts toward its mechanical behavior. In flip-chip packages, the underfill layer serves not only as a medium for transferring and redistributing thermomechanical stresses but also plays a critical role in governing crack initiation, crack propagation, and residual-stress accumulation. Its mechanical characteristics therefore have a direct impact on the long-term reliability of the package.

Unlike conventional epoxy composites, underfills operate within a three-dimensionally constrained environment defined by the die, interconnects, and substrate. Their mechanical performance is consequently governed not by a single parameter, but by the combined effects of elastic modulus, fracture toughness, and residual stress generated during curing. These properties respectively determine the load-bearing capability, resistance to crack propagation, and initial stress state of the underfill layer, while being strongly coupled through filler loading, crosslink density, interfacial adhesion, and curing conditions. Mechanical optimization of underfills should therefore be regarded as a multi-objective design problem rather than the independent maximization of any single property.

### 5.1. Regulation of Elastic Modulus and Thermomechanical Stress

Elastic modulus determines the capacity of the underfill layer to sustain and redistribute thermomechanical loads and is one of the most direct mechanical parameters governing solder-joint fatigue life. After curing, the underfill forms a continuous supporting layer that constrains the relative displacement between the die and substrate during thermal cycling, thereby redistributing stresses that would otherwise be concentrated primarily in the solder joints throughout the package structure. Accordingly, an appropriate increase in modulus can reduce inelastic deformation in solder joints and extend thermal-cycling lifetime. Excessively high modulus, however, is not necessarily beneficial. As the modulus increases, a greater fraction of the thermomechanical stress can be transferred from the solder joints to interfaces and low-k dielectric layers, potentially promoting interfacial delamination or die cracking when interfacial adhesion is insufficient. Therefore, the modulus should be optimized by balancing the effective constraint of solder-joint deformation against excessive stress transfer to mechanically vulnerable layers. The optimum modulus of an underfill is thus inherently dependent on the architecture and material configuration of the package. Lee et al. [52] employed finite-element analysis to investigate the effects of the elastic modulus of wafer-level underfill (WLUF) and die thickness on the thermal-cycling reliability of microbumps in three-dimensional integrated circuits (3D ICs). Their results showed that, for ultrathin stacked-die configurations, reducing the elastic modulus of WLUF could decrease the equivalent plastic strain in microbumps, thereby extending the predicted fatigue life. Die thickness also exerted a significant influence on package deformation and the stress state of the microbumps. These findings demonstrate that the mechanical properties of underfills cannot be optimized independently of the package architecture; instead, an appropriate mechanical-property window must be established according to the specific structural configuration and failure mechanisms of the package.

Beyond formulation composition, the curing process also plays an important role in determining the final modulus of underfill materials. Plachý et al. [53] investigated a SiO_2_-filled epoxy underfill composite and designed different isothermal curing schedules based on kinetic analysis. Although all curing routes produced nearly complete conversion, the resulting samples exhibited markedly different mechanical and thermomechanical properties as a function of curing temperature. Among the investigated conditions, the sample cured at 170 °C exhibited the highest modulus and strength. DMA measurements further indicated that different curing histories generated crosslinked networks with different degrees of structural uniformity. A narrower T_g_ peak was associated with a more homogeneous network and superior overall mechanical performance. These results demonstrate that, even at comparable levels of cure conversion, the temperature–time history of curing can substantially influence the final properties by altering network homogeneity and filler distribution. Consequently, curing-process optimization should be considered in conjunction with formulation design rather than treated as an independent processing step.

Meanwhile, underfills are typical viscoelastic polymer composites whose mechanical response continuously varies with temperature and loading frequency. Lall et al. [54] investigated the evolution of the viscoelastic behavior of epoxy molding materials through long-term high-temperature aging experiments and demonstrated that the mechanical response of packaging polymers is strongly affected by both temperature and service duration. Amagai et al. [55] characterized the viscoelastic properties of molding compounds and underfill materials using dynamic mechanical analysis (DMA) and thermomechanical analysis (TMA) to determine their viscoelastic moduli and coefficients of thermal expansion, respectively. Nanoindentation was further employed to obtain local mechanical parameters, and the resulting material properties were incorporated into finite-element models for prediction and experimental validation of package warpage. These studies demonstrate that the mechanical response of underfill materials exhibits pronounced time–temperature dependence and cannot be adequately represented by a single constant elastic modulus under actual service conditions. Mechanical characterization of underfills should therefore progress beyond conventional static-modulus measurements toward comprehensive characterization of viscoelastic parameters and their time–temperature dependence, thereby improving the accuracy of package-reliability assessments and warpage predictions.

### 5.2. Enhancement of Fracture Toughness and Regulation of Crack Propagation

Elastic modulus governs how stress is transferred within the material, whereas fracture toughness determines its resistance to failure under applied loading. A high crosslinking density endows epoxy underfills with high stiffness and dimensional stability but simultaneously restricts molecular-chain mobility, thereby limiting effective plastic energy-dissipation mechanisms. The incorporation of a high loading of inorganic fillers further increases the interfacial density, rendering filler–matrix interfaces preferential sites for crack initiation. Accordingly, enhancing fracture toughness has long been a key objective in the mechanical design of underfill materials.

In recent years, toughening strategies for underfill systems have increasingly focused on tailoring interfacial structures and regulating crack propagation pathways. Wu et al. [56] demonstrated that stronger interfacial bonding is not necessarily more beneficial by systematically varying the type and grafting density of silane coupling agents on the surface of SiO_2_. Excessively strong interfacial adhesion can make interfacial debonding energetically unfavorable, thereby suppressing crack deflection along the filler–matrix interface and favoring crack penetration into the resin matrix. In contrast, appropriately tailored interfacial interactions can promote controlled interfacial debonding, crack deflection, and branching, thereby increasing crack-path tortuosity and enhancing fracture energy dissipation. Thus, interfacial engineering should not simply maximize adhesion strength but rather establish an appropriate balance between effective load transfer and controlled interfacial fracture, such that sufficient interfacial integrity is maintained while energy-dissipating crack-deflection mechanisms can be activated. Zhang et al. [57] compared the effects of calcined and uncalcined SiO_2_ fillers and found that reducing the surface hydroxyl density improved interfacial compatibility while simultaneously enhancing fracture toughness and reducing moisture uptake. These findings indicate that the key to interfacial engineering lies not simply in maximizing interfacial bonding strength but in rationally tuning interfacial interactions to achieve a balance between interfacial stability and energy dissipation through crack deflection.

Beyond conventional macroscopic mechanical characterization, emerging in situ characterization techniques have provided new insights into the toughening mechanisms of underfill materials. Yu et al. [58] employed a coupled scanning electron microscopy and digital image correlation (SEM-DIC) approach to monitor the evolution of local strain fields near crack tips in different toughened systems. The results revealed distinct local deformation characteristics associated with different toughening mechanisms. Elastomer-toughened systems generated a relatively large plastic deformation zone near the crack tip, thereby dissipating more fracture energy and enhancing fracture toughness. In contrast, rigid nanofillers primarily retard crack propagation through mechanisms such as crack deflection, crack bridging, and crack pinning. These in situ characterization techniques provide direct experimental evidence for elucidating the structure–property relationships underlying the toughening of underfill materials.

**Figure 4:**
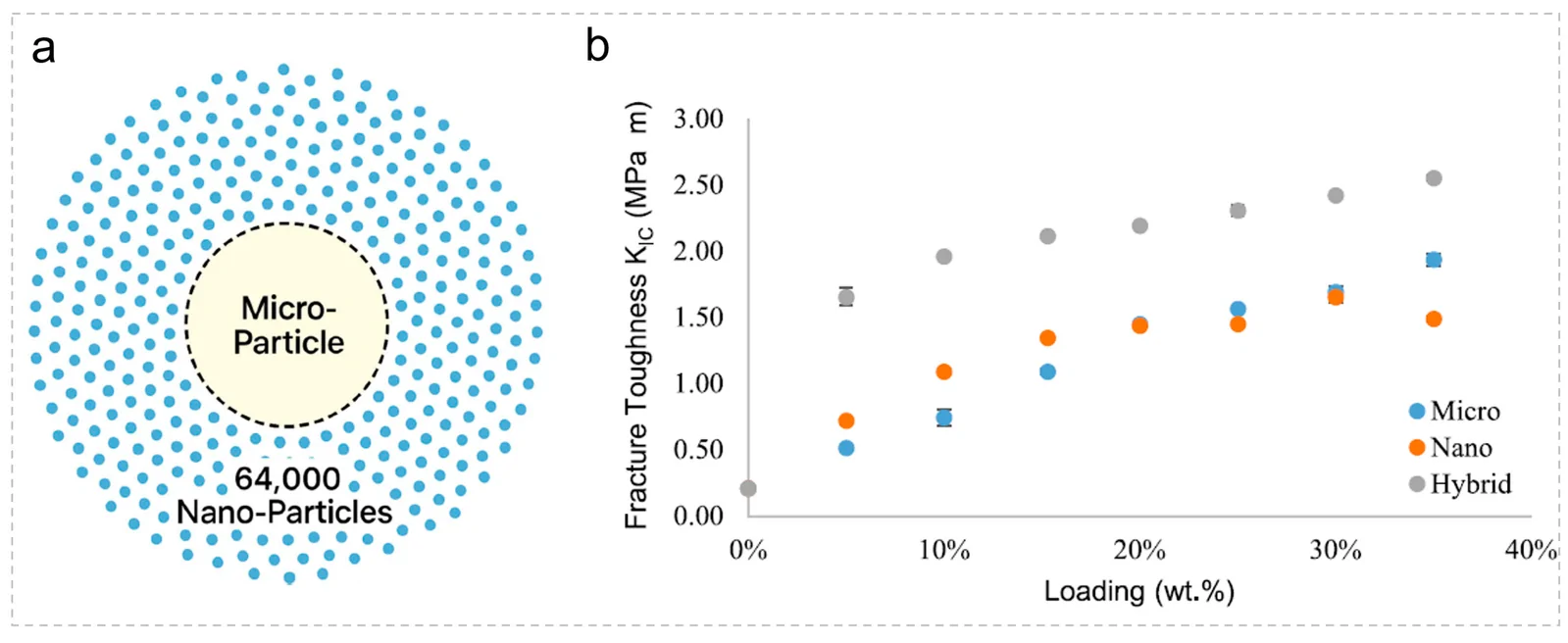
Synergistic toughening in hybrid micro/nano-silica filled epoxy: (**a**) schematic of the stress shielding effect, where one microparticle shields ~64,000 nanoparticles; (**b**) fracture toughness of micro-, nano-, and hybrid silica-filled epoxy vs. filler loading [59].

Building on these findings, synergistic design across different filler length scales offers a promising strategy for further regulating crack propagation and energy-dissipation mechanisms. Othman et al. [59] systematically investigated the fracture behavior of epoxy composites containing microscale SiO_2_, nanoscale SiO_2_, and their hybrid systems and demonstrated that fillers with different characteristic length scales can synergistically dissipate fracture energy through complementary toughening mechanisms. Microscale SiO_2_ primarily enhances toughness through crack pinning, crack deflection, and crack bridging, thereby increasing the tortuosity of crack propagation paths, whereas nanoscale SiO_2_ mainly promotes the formation of shear bands and localized void growth induced by interfacial debonding, thereby enhancing plastic energy dissipation. In the hybrid micro/nano-SiO_2_ system, the microscale particles can further modulate the local stress distribution through a stress-shielding effect, suppress excessive debonding of neighboring nanoparticles, and thereby help preserve interfacial integrity under high filler loadings (Figure 4a). Fracture toughness measurements further demonstrated that the hybrid system exhibited a marked improvement in fracture toughness compared with neat epoxy and consistently outperformed the corresponding single-scale filler systems across the entire filler-loading range (Figure 4b). Notably, the multiscale architecture also mitigated the toughness deterioration associated with nanoparticle agglomeration, thereby enabling synergistic toughening. This strategy is well aligned with the highly filled, multiscale filler design required for underfill materials and provides a promising pathway for enhancing fracture toughness while maintaining low CTE and high modulus.

Furthermore, increasing attention has been directed toward network toughening through molecular design of curing agents. For example, Zhang et al. [60] incorporated flexible siloxane segments into the curing-agent structure, substantially enhancing fracture toughness while maintaining a relatively high T_g_ and modulus. Unlike conventional rubber toughening, this strategy introduces flexible structural units directly into the crosslinked network to regulate molecular-chain mobility and stress-dissipation mechanisms, without requiring the incorporation of a large amount of a discrete toughening phase. It can therefore mitigate the adverse effects of conventional tougheners on resin viscosity and capillary filling, making it particularly attractive for underfill systems where processing flowability is critical.

Overall, toughening strategies for underfill materials are evolving from conventional single-mode interfacial strengthening or second-phase toughening toward a broader framework encompassing interfacial structure engineering, crack-path design, multiscale filler synergy, and molecular engineering of the curing network. Interfacial engineering primarily regulates crack initiation and deflection; multiscale fillers establish complementary energy-dissipation mechanisms across different length scales, while curing-network design introduces tailored molecular deformation and energy-dissipation capabilities at the molecular level. For highly filled underfill systems, future efforts should focus on elucidating and balancing the coupled relationships among fracture toughness, modulus, CTE, and viscosity, while integrating multiscale filler architectures with interfacial chemistry and curing-network design to achieve simultaneous optimization of mechanical performance and processability.

### 5.3. Regulation of Cure-Induced Internal and Residual Stresses

Unlike elastic modulus and fracture toughness, cure-induced internal stress is not an intrinsic material property in the conventional sense; nevertheless, it determines the initial stress state of the package and persists throughout subsequent thermal cycling, hygrothermal aging, and mechanical loading. Accordingly, increasing attention has been devoted to cure-induced stress as a third critical mechanical parameter governing underfill reliability. In underfill materials, residual stress primarily originates from two contributions: chemical cure shrinkage and thermally induced mismatch. These two processes evolve continuously over different stages of the thermal history and become superimposed within the constrained package structure, ultimately determining the magnitude of package warpage and interfacial stresses.

Chemical cure shrinkage arises from the reduction in intermolecular spacing as the epoxy system transforms from a low-viscosity liquid or oligomeric state into a highly crosslinked network. Plepys and Farris initially demonstrated from a theoretical perspective that, under unconstrained conditions, volumetric contraction can be accommodated through free shrinkage [61]. In an underfill layer, however, shrinkage is strongly constrained by the surrounding chip and substrate, preventing free dimensional contraction and consequently converting part of the volumetric shrinkage into internal elastic strain and cure-induced stress. During subsequent cooling from the cure temperature to ambient temperature, additional thermomechanical stress develops because of the substantial CTE mismatch between the underfill, silicon chip, and organic substrate. Thus, the final residual stress state should be regarded as the outcome of the coupled evolution of cure-shrinkage-induced stress and thermally induced stress, rather than as a consequence of a single mechanism.

Because the underfill layer is typically only several tens of micrometers thick and is confined between the chip and substrate, conventional strain-measurement techniques are often inadequate for directly resolving its internal stress evolution. Recent advances in in situ characterization have therefore provided new experimental approaches for elucidating the development of cure-induced stress. Peng et al. [62] established a real-time measurement platform for monitoring cure-induced shrinkage in underfill systems and systematically investigated the effects of filler composition, coupling agents, and toughening agents on shrinkage behavior, thereby establishing correlations between formulation parameters and cure shrinkage. Subsequently, Peng et al. [63] performed in situ characterization of cure-induced residual stress under three-dimensional constraint using a flip-chip test vehicle (FCTV). Their results revealed that shrinkage within an actual package is not isotropic but exhibits pronounced directional dependence owing to the three-dimensional mechanical constraints. In particular, the shrinkage in the Z direction normal to the chip surface was substantially greater than that in the X/Y directions and made a dominant contribution to package warpage. This finding extends the conventional interpretation of package deformation based primarily on CTE mismatch by demonstrating that anisotropic cure shrinkage should also be incorporated into the reliability analysis and mechanical design of underfill materials.

Beyond experimental measurements, multiphysics finite element (FE) simulation has become an important tool for analyzing residual stresses in underfill-containing packages. Zhong et al. [64] developed an FE model incorporating cure kinetics and thermal expansion behavior, enabling the separate quantification of cure-shrinkage-induced stress and thermally induced mismatch stress and thereby providing a theoretical basis for identifying the respective contributions of different stress sources in various material systems. Li et al. [65] further incorporated viscoelasticity into simulations covering the entire packaging process and continuously analyzed underfill curing, molding, and reflow soldering in 2.5D advanced packages. Their results showed that stress relaxation within the polymer network at elevated temperatures can partially offset the warpage induced by cure shrinkage, indicating that neglecting viscoelastic effects can substantially overestimate residual stress in the package.

From a materials-design perspective, the mechanical properties of underfills require a dynamic balance among stiffness, toughness, and residual stress. Increasing elastic modulus can effectively suppress plastic deformation of solder joints, but excessive stiffness may intensify stress concentration at interfaces. Improving fracture toughness can retard crack propagation, yet conventional toughening approaches often involve reduced crosslink density or increased formulation viscosity. Meanwhile, residual stresses arising from cure shrinkage and thermal mismatch persist across material processing, package assembly, and long-term service, continuously affecting package warpage and interfacial reliability.

Advances in finite element simulation, in situ strain characterization, and viscoelastic modeling have progressively shifted underfill mechanical research from empirical formulation optimization toward mechanism-driven materials design. Nevertheless, compared with thermal-property studies, the mechanical behavior of underfills remains relatively underexplored. In highly filled systems in particular, systematic understanding of crack-propagation mechanisms, interfacial failure, and residual-stress evolution remains limited. Most existing studies continue to focus on individual mechanical parameters, while a unified design framework that integrates the coupling among different properties and their compatibility with specific package architectures has yet to be established.

## 6. Progress in Reliability and Integrated Performance

The thermal, rheological, and mechanical properties of underfill materials govern their heat dissipation capability, processing window, and structural support, respectively; however, these individual parameters do not directly translate into long-term service performance. In flip-chip packages, the cured underfill is continuously exposed to multiple service conditions, including thermal cycling, hygrothermal environments, and mechanical loading. Its reliability is therefore governed not only by the intrinsic properties of the material but also by the stability of the filler–resin interface and the ongoing evolution of cure-induced residual stresses. In this regard, underfill reliability is essentially a multidimensional manifestation of thermal, rheological, and mechanical properties under service conditions. Different failure pathways may be dominated by different performance attributes, and the effects of material modification on reliability must therefore be assessed from an integrated perspective.

### 6.1. Effect of Filler Engineering on the Service Reliability of Underfill Materials

Changes in filler loading, spatial distribution, and filler type simultaneously modify the CTE, Young’s modulus, thermal conductivity, and density of filler–matrix interfaces. These parameters collectively influence hygrothermal and thermal-cycling reliability through distinct mechanisms and exhibit pronounced nonlinear coupling. Regarding the effect of filler loading on reliability, Kim et al. [66] systematically investigated the influence of different Al_2_O_3_ contents on the thermomechanical properties and reliability of BGA underfill materials and evaluated package reliability using thermal-shock and three-point bending tests. Increasing the Al_2_O_3_ content reduced the CTE while increasing the material modulus and thermal conductivity. Nevertheless, optimization of filler loading required a balance between the intrinsic material properties and the resulting package-level reliability. The spatial distribution of fillers is also critical. Suzuki et al. [67] demonstrated that local nonuniformity in filler-particle distribution can substantially accelerate delamination at the polyimide (PI)/underfill interface. By extracting filler-distribution characteristics through image processing and incorporating them into finite element analysis, the location of interfacial failure under thermal cycling could be quantitatively predicted. These findings indicate that controlling filler homogeneity can be as important as optimizing the overall filler content.

The influence of fillers on hygrothermal reliability is likewise significant. Bi et al. [68] combined in situ SEM-DIC characterization with positron annihilation measurements to systematically investigate the effect of filler content on the hygrothermal aging behavior of epoxy underfills under 121 °C/100% relative humidity conditions. After hygrothermal aging, cracks preferentially initiated along filler–matrix interfaces, while increasing filler content resulted in increasingly pronounced interfacial-debonding-dominated failure. Although strong filler–matrix interfaces are desirable for effective stress transfer, higher filler contents also increase the total interfacial area, thereby increasing the number of potential sites for interfacial debonding and crack initiation. These observations demonstrate that although high filler loading can improve thermal properties, it simultaneously increases the overall population of interfaces susceptible to environmental degradation.

With respect to reliability prediction and optimization, Lim et al. [69] combined finite element analysis with design-of-experiments approaches to optimize the nonlinear effects of underfill thickness and solder content on chip stress, thereby identifying an optimal combination of design parameters. Pan and Zou [70] further employed ANSYS simulations to investigate the effect of underfill T_g_ on the thermal-fatigue lifetime of SAC305 solder joints, showing that a higher underfill T_g_ was associated with a longer predicted thermal-fatigue lifetime and thus providing quantitative guidance for underfill material selection.

Overall, filler engineering influences reliability through multiple interconnected pathways. Filler loading governs CTE and modulus but simultaneously constrains processability through viscosity; filler distribution affects the rate and localization of interfacial degradation; and T_g_ governs mechanical stability under elevated-temperature service. Consequently, optimal filler design requires a balance among thermal transport, processability, and interfacial durability. Quantitative assessment of this balance still relies heavily on package-level simulations, while a unified predictive framework capable of linking filler characteristics to long-term reliability remains to be established.

### 6.2. Effect of Resin Matrix and Interfacial Modification on the Reliability of Underfill Materials

Hygrothermal aging is one of the critical factors governing the long-term reliability of epoxy-based underfill materials. The degradation process generally involves moisture diffusion into the epoxy network and filler–matrix interfaces, plasticization of the epoxy matrix, and progressive deterioration of interfacial adhesion. Moisture uptake can not only reduce the glass transition temperature and mechanical properties of epoxy materials but may also preferentially accumulate at relatively hydrophilic interfaces, thereby accelerating interfacial degradation. For underfill materials, He et al. [9] investigated moisture absorption and hygroscopic swelling and demonstrated that moisture uptake can induce pronounced hygrothermal expansion, which subsequently affects the dimensional stability and reliability of the package.

At the level of bulk-material design, Seo et al. [71] developed a dual-network epoxy composite by incorporating poly(methyl methacrylate)-*b*-poly(2-(dimethylamino)ethyl methacrylate) (PMMA-*b*-PDMAEMA, PMD) as a block-copolymer compatibilizer. The incorporation of PMD increased the crosslink density of the epoxy/SiO_2_ system and strengthened the filler–matrix interfacial interactions, thereby substantially suppressing moisture uptake. The resulting composite exhibited reduced water absorption and enhanced tensile modulus.

**Figure 5:**
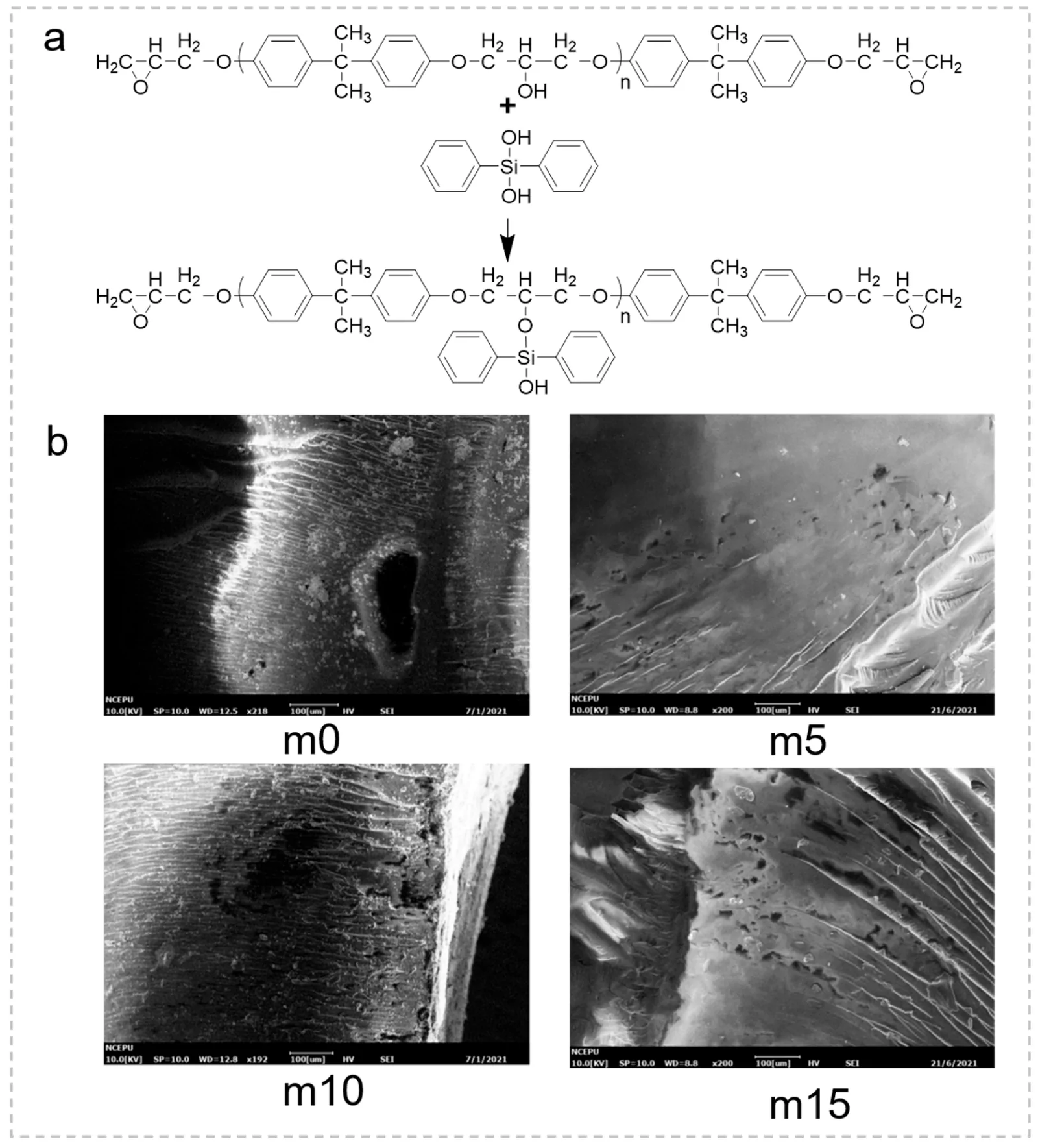
(**a**) Grafting reaction of diphenylsilanediol onto bisphenol A-based epoxy molecular chains. (**b**) Cross-sectional micromorphology of the samples after 16 days of aging: m0, m5, m10, and m15 [72].

Beyond increasing the density of the crosslinked network, hydrophobic modification of the matrix chemical structure represents another effective strategy for regulating the bulk moisture resistance of epoxy underfills. Wang et al. [72] grafted diphenylsilanediol onto the molecular chains of bisphenol A-based epoxy resin (Figure 5a). Hygrothermal aging experiments showed that, when the modification content was below 10 wt%, the fitted equilibrium water uptake decreased from 1.08% to 0.61%. Further increasing the modification content, however, led to a rebound in water uptake, which was attributed to the presence of unreacted low-molecular-weight species. Cross-sectional morphological observations further confirmed this compositional dependence (Figure 5b). After aging, the moderately modified samples (m5 and m10) exhibited only relatively small cracks with characteristic dimensions of approximately 20–30 μm, whereas the unmodified sample (m0) developed pronounced large voids. At excessive modification levels (m15), these voids tended to coalesce into elongated cracks, consistent with the observed variation in water uptake. These results demonstrate that hydrophobic molecular modification can effectively improve the moisture resistance of epoxy underfills, but its effectiveness strongly depends on the modification level, highlighting the importance of identifying an appropriate compositional window. Wang et al. [73] employed molecular dynamics simulations to investigate the effects of different silane coupling agents on the hygrothermal aging resistance of epoxy/SiO_2_ interfaces. The results showed that silane coupling agents increased the interfacial binding energy and enhanced interfacial stability. Amino-functionalized silanes provided stronger stabilization than epoxy-functionalized silanes because of their greater contribution from covalent interactions. Meanwhile, the molecular structure of the silane coupling agent affected the local polymer density at the interface and consequently the accessibility of water molecules to the interfacial region.

Studies focusing on the underfill/passivation-layer interface have likewise demonstrated that interfacial hydrophilicity is an important factor governing adhesion retention after hygrothermal aging. Relatively hydrophilic SiO_2_ and Si_3_N_4_ surfaces exhibited more pronounced degradation in adhesion, whereas the introduction of silane coupling agents to establish stable interfacial chemical bonds effectively improved interfacial stability under hygrothermal conditions [74]. Overall, the hygrothermal reliability of underfill materials cannot be determined solely by their bulk water-absorption characteristics. Instead, it is governed by the coupled effects of moisture transport, polymer-network structure, and filler–matrix interfacial stability. Accordingly, mitigation strategies are evolving from simply reducing matrix hygroscopicity toward increasing network compactness and constructing more stable interfacial chemical bonds. The underlying objective is to reduce the accessibility of moisture within both the polymer matrix and interfacial regions while suppressing moisture-induced degradation of interfacial adhesion.

Regarding the effects of toughening modifications on cure-induced stress and reliability, Yang et al. [75] systematically compared three types of rubber tougheners—methyl methacrylate–acrylate–methyl methacrylate (MAM-R) block rubber, polybutadiene rubber (PBd-R), and polydimethylsiloxane (PDMS-R)—and evaluated their effects on cure-shrinkage-induced stress in underfills for 2.5D packages. Among the three systems, PBd-R-modified underfill achieved the most favorable balance between mechanical reinforcement and processability, whereas the MAM-R- and PDMS-R-based systems required further formulation optimization. This study demonstrates that although toughening agents can improve fracture resistance, they can simultaneously alter cure-shrinkage behavior and residual-stress distribution. Their overall effect therefore depends strongly on the compatibility between the toughening mechanism and the epoxy matrix.

In addition, Yang et al. [76] developed an artificial neural network (ANN)-based model for predicting underfill delamination lifetime and combined it with extended finite element method (XFEM) simulations to characterize crack-propagation paths. By systematically evaluating the effects of five manufacturing variables on underfill delamination and cracking, the model achieved close agreement with experimental observations, providing a data-driven approach for quantitatively assessing the effects of interfacial modifications. This study illustrates the potential of machine learning to establish nonlinear relationships between multiple manufacturing variables and package-level reliability outcomes. When combined with physics-based simulations such as XFEM, data-driven models can further connect formulation and process variables with failure behavior, providing a potential pathway from empirical reliability evaluation toward predictive optimization.

Overall, the influence of resin-matrix and interfacial modifications on underfill reliability can be broadly understood through three interconnected pathways: suppression of moisture uptake, strengthening of interfacial bonding, and regulation of cure-induced stress. The integration of data-driven modeling further enables the effects of material modifications to progress from empirical screening toward quantitative structure–process–reliability correlations.

### 6.3. Optimization of Curing Systems and Reliability Design for Advanced Packaging

Package warpage originates primarily from the accumulation of residual stresses generated by cure shrinkage when viscoelastic stress relaxation is incomplete. The resulting reliability concerns include interfacial delamination, solder-joint cracking, and subsequent assembly failure. In contrast to the molecular-level mechanisms of cure shrinkage discussed earlier, this section focuses on the package-level consequences of curing behavior, particularly its influence on warpage evolution and long-term reliability. Zhou et al. [77] employed a generalized Maxwell model to investigate the effect of viscoelastic evolution during underfill curing on the warpage of fan-out packages. The results showed that package warpage changed substantially once the degree of cure exceeded 90%. During subsequent cooling, the warpage continuously decreased, crossed zero, and then increased in the opposite direction, ultimately resulting in reversed warpage at room temperature. This behavior can be attributed to the competition between viscoelastic stress relaxation and CTE-mismatch-induced thermal contraction during cooling: stress relaxation progressively releases the residual stresses accumulated during curing, whereas differential thermal contraction between the underfill and adjacent package materials drives warpage in the opposite direction. When the balance between these effects changes during cooling, the warpage can pass through zero and subsequently reverse its direction. These findings demonstrate that the relationship between the degree of cure and warpage evolution is far from linear and is strongly influenced by the coupled effects of cure progression, thermal contraction, and viscoelastic stress relaxation. Liu et al. [78] introduced a virtual-material approach that uses equivalent material parameters to replace conventional Prony-series representations, substantially improving the computational efficiency of viscoelastic finite element simulations. By further incorporating cure shrinkage and viscoelasticity into a coupled warpage-prediction framework, the approach was validated for image-sensor packaging and showed good agreement with experimental results. This methodology provides an efficient simulation tool for evaluating and controlling warpage under different manufacturing process paths.

In large-scale advanced packages, the effects of underfill material parameters on warpage become increasingly complex. Li et al. [79] developed a finite element model of a 66 mm × 84 mm large-area multichip package containing approximately 13,000 solder balls and systematically investigated the effects of underfill T_g_, Young’s modulus, CTE, and curing temperature on global package warpage. The results showed that package warpage decreased with decreasing underfill Young’s modulus and curing temperature, whereas the effects of T_g_ and CTE were comparatively limited. These findings provide a useful parameter hierarchy for underfill selection in large-scale packaging.

With the rapid development of chiplet and 2.5D integration, reliability challenges are increasingly extending beyond the optimization of individual material parameters. Kim et al. [80] systematically reviewed the thermomechanical challenges associated with 2.5D packaging and identified package warpage induced by large-area silicon interposers and board-level interconnect fatigue as key reliability bottlenecks. Their analysis further highlighted the strong coupling among material properties, package geometry, and manufacturing process conditions. Chou et al. [81] evaluated the reliability of a chiplet-based heterogeneous 2.3D hybrid-substrate package through thermal cycling and drop tests. Weibull analysis was employed to characterize solder-joint failure modes, while a linear acceleration factor was used to extrapolate the reliability measured under accelerated test conditions to actual service conditions, providing a standardized approach for reliability assessment of advanced packages.

Overall, reliability-oriented underfill research is increasingly centered on multidimensional performance optimization. Filler engineering requires a balance between enhanced thermal transport and long-term interfacial durability; resin-matrix and interfacial modifications improve hygrothermal reliability through moisture suppression and stress regulation; and curing-system optimization provides a means of controlling package warpage and residual stress at their source. The coupled effects of these three aspects ultimately determine the long-term performance of advanced packages. Establishing quantitative relationships among material composition, interfacial structure, process history, and package lifetime, supported by physics-based simulations and machine-learning-assisted data-driven models, will be critical for advancing underfill reliability from empirical evaluation toward predictive design.

## 7. Conclusions

After decades of development, epoxy underfill materials have evolved into a multilevel design system encompassing resin molecular structure, filler architecture, interfacial engineering, and curing processes. In this review, recent advances in the key performance requirements of underfill materials—including thermal, rheological, mechanical, and reliability characteristics—have been systematically summarized. Overall, underfill design is progressively shifting from the optimization of individual properties toward the coordinated regulation and matching of multiple performance attributes. Thermal-property engineering has evolved from simply reducing the coefficient of thermal expansion (CTE) toward the simultaneous optimization of low CTE and high thermal conductivity, with particle-size distribution, structured fillers, and hybrid filler networks being employed to improve filler utilization efficiency. Rheological regulation primarily addresses the flow and processing requirements of highly filled formulations, in which the molecular structure of the resin, particle packing, and interfacial interactions are jointly tailored to reduce viscosity while retaining adequate filling capability. Mechanical design has likewise progressed beyond individual modulus or toughness targets toward a balance among stiffness, fracture resistance, and residual stress, while viscoelastic characterization and curing-process simulations have provided new tools for understanding and controlling package-level stress. Ultimately, however, material performance must be validated under realistic service conditions, including thermal cycling, hygrothermal aging, and package warpage. This highlights that the overall performance of an underfill is governed by the coupled effects of the polymer network, filler architecture, interfacial state, and curing history.

Looking ahead, several areas warrant further attention to meet the evolving requirements of advanced packaging. First, cross-scale structure–property relationships should be further established to quantitatively link molecular structure, filler and interfacial characteristics with macroscopic rheological and thermomechanical properties—and, ultimately, package-level reliability. Such correlations are essential for enhancing the predictability of materials design. Second, application-specific underfill systems should be developed for emerging packaging architectures, including chiplet, 2.5D/3D integration, large-die packages, and high-power devices, with judicious trade-offs among low stress, rapid filling, high thermal conductivity, and low dielectric loss. Third, high-throughput experimentation, machine learning, and data-driven approaches can be integrated with conventional materials development to accelerate formulation screening and multi-objective optimization. Their practical implementation, however, will depend critically on the availability of high-quality, standardized, and comparable materials- and package-level datasets. Establishing consistent reporting and evaluation practices for key material properties, processing conditions, and package-level reliability metrics will therefore be an important prerequisite for future data-driven underfill development. In addition, low-temperature rapid curing, energy-efficient manufacturing, and reworkable underfill materials represent promising directions for reducing the energy consumption and resource requirements associated with advanced packaging. In addition, sustainability and reworkability should receive greater attention in future underfill design. Low-temperature rapid curing and energy-efficient manufacturing can reduce the energy demand associated with package fabrication, while reworkable underfills may facilitate component replacement and reduce material waste during package repair and end-of-life processing. Thus, future underfill development should increasingly consider energy consumption, resource efficiency, and reworkability alongside conventional performance and reliability requirements.

Overall, the future development of underfill materials should move beyond maximizing any single performance metric and instead focus on achieving rational compatibility among material composition, microstructure, processing conditions, package architecture, and service environment. As advanced packaging continues to evolve toward higher density, greater power dissipation, and increasingly heterogeneous integration, the establishment of an application-oriented materials design methodology—one that integrates structure–property relationships, process optimization, and reliability validation—will be essential for the next generation of high-performance epoxy underfill materials.

## Data Availability

No new data were created or analyzed in this study. Data sharing is not applicable to this article.
